# Understanding Inequalities in Mobile Health Utilization Across Phases: Systematic Review and Meta-Analysis

**DOI:** 10.2196/71349

**Published:** 2025-08-14

**Authors:** Seongwoo Yang, Myoung Jin Cha, Robin van Kessel, Govind Warrier, Johannes Thrul, Mangyeong Lee, Junghee Yoon, Danbee Kang, Juhee Cho

**Affiliations:** 1 Department of Digital Health, SAIHST, Sungkyunkwan University, Seoul, Republic of Korea; 2 Center for Clinical Epidemiology, Samsung Medical Center, Sungkyunkwan University School of Medicine, Seoul, Republic of Korea; 3 LSE Health, Department of Health Policy, London School of Economics and Political Science, London, United Kingdom; 4 Department of International Health, Care and Public Health Research Institute (CAPHRI), Maastricht University, Maastricht, The Netherlands; 5 Sidney Kimmel Comprehensive Cancer Center, Department of Oncology, Johns Hopkins University School of Medicine, Baltimore, MD, United States; 6 Bloomberg-Kimmel Institute for Cancer Immunotherapy, Johns Hopkins University, Baltimore, MD, United States; 7 Department of Mental Health, Johns Hopkins Bloomberg School of Public Health, Baltimore, MD, United States; 8 Centre for Alcohol Policy Research, La Trobe University, Melbourne, Australia; 9 Department of Clinical Research Design and Evaluation, SAIHST, Sungkyunkwan University, 115 Irwon-ro, Gangnam-gu, Seoul, 06355, Republic of Korea; 10 Institute for Quality of Life in Cancer, Samsung Medical Center, Seoul, Republic of Korea

**Keywords:** mobile health, inequalities, digital divide, social determinants of health, digital health, mobile phone

## Abstract

**Background:**

Mobile health (mHealth) holds promise for enhancing patient care, yet attrition in its use remains a major barrier. Low retention rates limit its potential impact, while barriers to accessing or adopting mHealth vary across populations and countries. These differences in utilization of mHealth may exacerbate health inequalities, contributing to the digital health divide.

**Objective:**

We aimed to conduct a systematic review and meta-analysis to investigate the factors associated with inequalities in mHealth utilization across different implementation phases, including access, adoption, adherence, and maintenance.

**Methods:**

This systematic review and meta-analysis analyzed mHealth research from 2000 to May 30, 2024, using databases, including PubMed, Web of Science, MEDLINE, and ProQuest. Eligible studies included smartphones, mHealth apps, wearables, and inequality indicators across 4 mHealth phases: access, adoption, adherence, and maintenance. Excluded studies were nonpeer-reviewed, opinion-based, or not in English. Extracted data included study characteristics, target populations, health outcomes, and inequality factors like age, gender, socioeconomic status, and digital literacy. Factors were categorized using a digital health equity framework (biological, behavioral, sociocultural, digital, health care system, and physical domains). Meta-analyses were performed using a random-effects model for factors reported in at least three studies, with heterogeneity assessed by the *I*² statistic.

**Results:**

Among 1990 studies, 62 studies met the inclusion criteria, and 30 studies underwent meta-analysis. The phases of mHealth utilization were access (n=23, 37%), adoption (n=47, 76%), adherence (n=9, 15%), and maintenance (n=2, 3%). Meta-analysis showed older age was negatively associated with mHealth adoption (odds ratio [OR] 0.47, 95% CI 0.23‐0.93), while higher education and income were positively associated in both access and adoption phases. Employment showed significant associations in the access phase (OR 1.49, 95% CI 1.08‐2.05), whereas comorbidities (OR 1.39, 95% CI 1.03‐1.86) and private insurance (OR 1.63, 95% CI 1.07‐2.48) were significantly associated with adoption of mHealth. Women (OR 1.24, 95% CI 1.06‐1.45) and physically active individuals (OR 1.64, 95% CI 1.07‐2.50) were more likely to adopt mHealth.

**Conclusions:**

The conceptual framework outlined in this study highlights the multifaceted nature of mHealth utilization across all the phases of mHealth engagement. To address these inequalities, tailored and personalized interventions are required at each phase of mHealth utilization. Targeted efforts can enhance digital access for older and low-income adults while promoting engagement through education, insurance support, and healthy behaviors, thereby promoting equitable and effective mHealth use. By recognizing the interconnectedness of these domains, policy makers and health care stakeholders can design interventions that not only address the phase-specific barriers but also bridge broader inequalities in health care access and engagement.

## Introduction

Mobile health (mHealth) apps constitute a major source of health information, health care decision-making, and health communication [1,2]. Estimates indicate that more than 350,000 mHealth apps are accessible on various mobile platforms [3-5], which can reach numerous people extensively, as internet use and smartphone ownership become common [6], despite uncertain quality and efficacy due to the unregulated free market [7]. Moreover, the recent COVID-19 pandemic has resulted in increased utilization of various mHealth apps [8,9], and mHealth has been used for a wide range of health management purposes, including HIV prevention, smoking cessation, and self-management of diabetes and depression [10-13]. Research has revealed that mHealth interventions can be as effective as face-to-face interventions in increasing physical activity [14,15] and reducing sedentary behavior [16]. Additionally, the use of artificial intelligence in mHealth apps is emerging to aid both individuals and health care professionals in the prevention and management of chronic diseases in a person-centered way [17].

Despite the promising potential of mHealth, a major barrier to patient care remains, namely, attrition in the use of mHealth interventions [18]. An observational study of app use in a large, real-world cohort of nearly 200,000 users worldwide found that only 2% had maintained continuous engagement [19]. These low retention rates suggest that the actual benefit of mHealth may be limited [20]. While clinical trials for mHealth interventions often report retention rates of 70% or higher, these trials are typically short-term, some lasting fewer than 2 months, and are unlikely to reflect real-world use [21]. Additionally, many individuals face barriers to accessing or adopting mHealth for health management, and these barriers vary significantly by country and target population [22,23]. Specifically, mHealth utilization is associated with demographic characteristics (age, gender, education level, and socioeconomic status) and health-related knowledge and management [24], as well as use of one’s smart devices [25], eHealth literacy, privacy concerns [26], social contexts [27], and patients and clinicians’ perspective on the value of mHealth apps [28]. Thus, it has been proposed that mHealth interventions could potentially widen health inequalities as part of the digital health divide [29]. However, challenges were notably found in low-resource regions, including cost, poor interactivity, lack of training, low acceptability, and misalignment with local funders [30,31]. Nontechnical issues like ethics, policy, equity, resource gaps, and evidence quality also posed barriers in the low- and middle-income countries [31].

The World Health Organization European Region attempted to classify equity within digital health technology into access, use, and engagement. However, these categorizations do not fully explain the exact definitions of each phase and do not include inequalities in mHealth utilization [32]. Furthermore, there is no universally accepted framework explaining the phases of mHealth utilization or how related factors interact to produce better clinical or behavioral outcomes. Therefore, we aimed to conduct a systematic review and meta-analysis to investigate the factors associated with inequalities in mHealth utilization across different implementation phases, including access, adoption, adherence, and maintenance. We also sought to develop a conceptual framework outlining the necessary components, relationships, and practical considerations across various domains. To our knowledge, this is the first systematic review and meta-analysis to comprehensively describe inequality indicators in each phase of mHealth implementation.

## Methods

### Search Strategies

The search for this study was performed based on the standards described in the PRISMA (Preferred Reporting Items for Systematic Reviews and Meta-Analyses) guidelines [33], and the protocol was registered with PROSPERO (ID: CRD42023466850) and has not been amended. The following databases were searched: PubMed, Web of Science, MEDLINE, and ProQuest. The search dates were limited to studies published in and after 2000 and up to May 30, 2024, because of the scarcity of studies. The keywords for the search strategy were primarily derived from MeSH, and the entry terms are listed (Multimedia Appendix 1). No additional studies were included after screening the reference lists of eligible studies.

### Study Selection

We included studies that defined mHealth with participants using smartphones, mHealth apps, digital therapeutics, wearables, and having inequality indicators related to mHealth across different implementation phases. Implementation of mHealth utilization was classified into four phases: access, adoption, adherence, and maintenance (Table 1) [32,34,35]. Studies were excluded if they (1) were reviews, commentaries, opinions, clinical trial protocols, or app development papers; (2) had no user engagement; (3) used face-to-face or other digital tools, such as computers or websites; (4) were not peer-reviewed; or (5) were not written in English due to language barriers. After removing duplicates, one author (SY) screened the titles and abstracts of all studies using the Rayyan AI platform. Then, two authors (SY and MJC) reviewed the full texts of the screened studies for final inclusion. Any disagreements were resolved through discussion or by the third author (JC).

**Table 1.** Definition of each implementation phase in mHealth^a^ utilization.

Phase	Definition	Example	Reference
Access	Users’ ability and availability to access the resources required for mHealth	Ownership of smartphones or wearables	[32]
Adoption	mHealth adoption determined by users or recommended by clinicians	Use of mHealth apps and digital health tools, downloads of health apps for diabetes	[34]
Adherence	Appropriate use of mHealth, whether prescribed or not, as directed	Engagement with mHealth or mobile, and continuing to use the app for at least 6 months	[34]
Maintenance	Continuous use of mHealth for a desirable period	Maintain the use of mHealth apps or wearables over 6 months	[35]

a mHealth: mobile health.

### Data Extraction

Data extraction was conducted by one author (SY) using the following predefined variables: first author, year, setting, type of study, target outcomes, population, health condition, sample size, mean age, phase of mHealth use (access, adoption, adherence, and maintenance), level of influence, type of intervention, mode of delivery, and type of estimate. Information on the use of mHealth at multiple time points and the average rate of mHealth utilization was also extracted. Inequality indicators for using mHealth included age, gender, socioeconomic position (including occupation, income, and employment), education level, health service accessibility, geographical indicators, sexual orientation, health literacy, and digital literacy. Measures of effects, such as odds ratios (ORs), prevalence ratios, and hazard ratios, were collected to aggregate the effect size of these indicators, if available.

### Quality Assessment

The quality of the studies was assessed using the Mixed Methods Appraisal Tool, which evaluates qualitative, quantitative, and mixed methods based on specific methodological criteria, with two authors independently conducting the assessment (SY and MJC) [36]. A consensus meeting was held to compare notes from the selected papers used in this review. An agreement was reached regarding these conflicting points.

### Data Synthesis and Analysis

The primary outcome of this study was mHealth utilization in the implementation phase (access, adoption, adherence, and maintenance). The clinical outcomes were also considered. For example, when changes in clinical outcomes for diabetes, such as HbA_1c_, varied according to specific indicators after the use of mHealth for a period, these were also deemed outcomes indicating inequalities in mHealth utilization. All accrued inequality indicators were classified into domains of influence, which were partially used from the framework for digital health equity (biological, behavioral, sociocultural, digital or mobile environment, health care system, and physical environment) [37]. The grouped factors were then presented as a framework.

### Meta-Analysis

Meta-analyses of eligible factors were performed when inequality factors were found in three or more studies with relevant outcomes, including the OR. Studies using measures other than the OR, such as the hazard ratio or prevalence ratio, were excluded. The inverse variance method was used for pooling. Studies with an effect size determined by other methods, such as regression analysis, factor analysis, or structural equation modeling, were excluded from the meta-analysis owing to the insufficient number of studies. A random-effects model was used to calculate the combined estimates of the overall effects, along with 95% CIs for all measures of effect. The *I^2^* statistic was used to assess discrepancies among studies (*I^2^*=0%‐100%; values>50% indicated significant statistical heterogeneity), and restricted maximum likelihood was used to synthesize each effect. Funnel plots were created to assess publication bias, and the presence of asymmetries or missing data sections was visually examined for meta-analyses in the access and adoption phases. Data were analyzed using R software (version 4.2.2; R Foundation for Statistical Computing).

## Results

### Selected Studies

**Figure 1.**
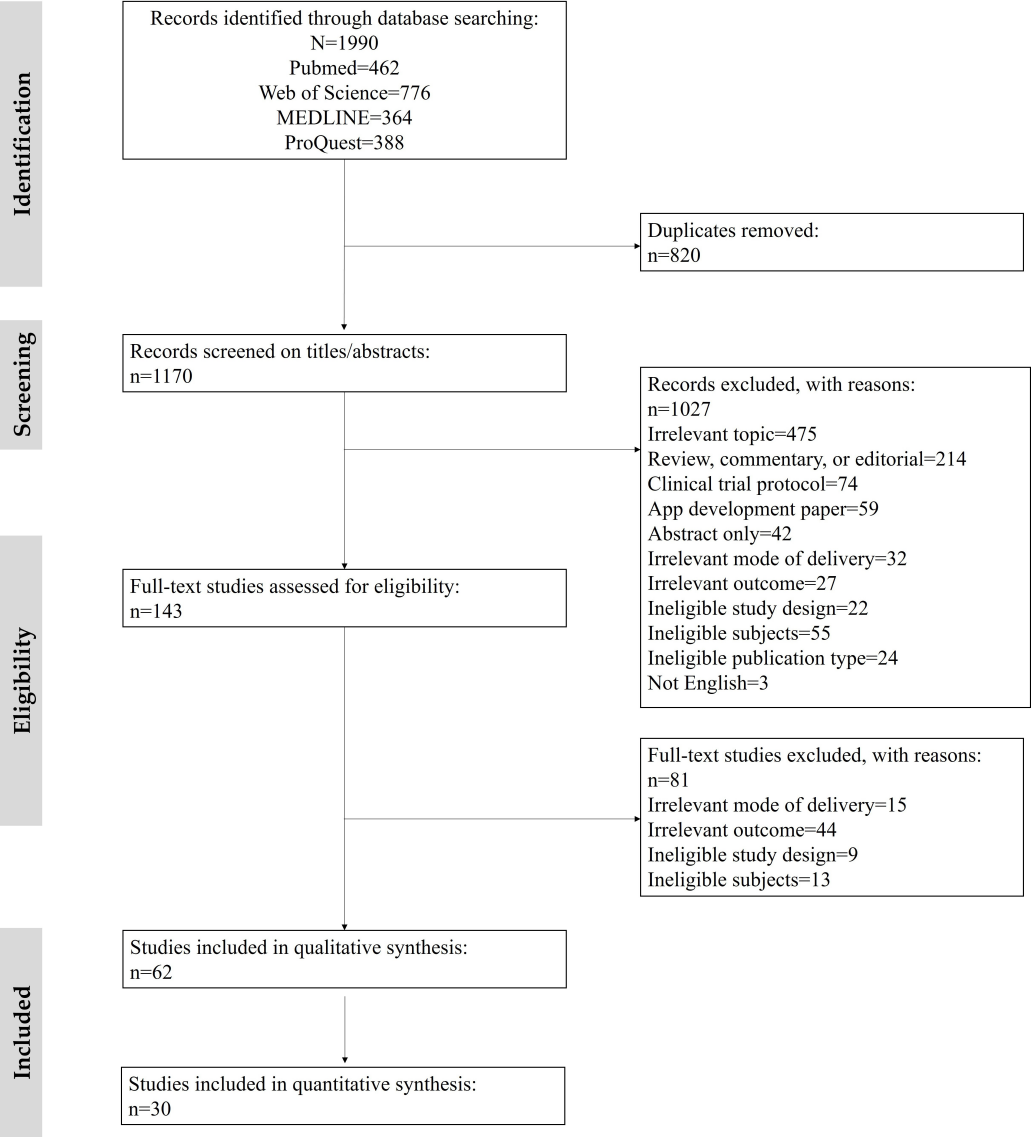
Study selection in the systematic review.

**Figure 2.**
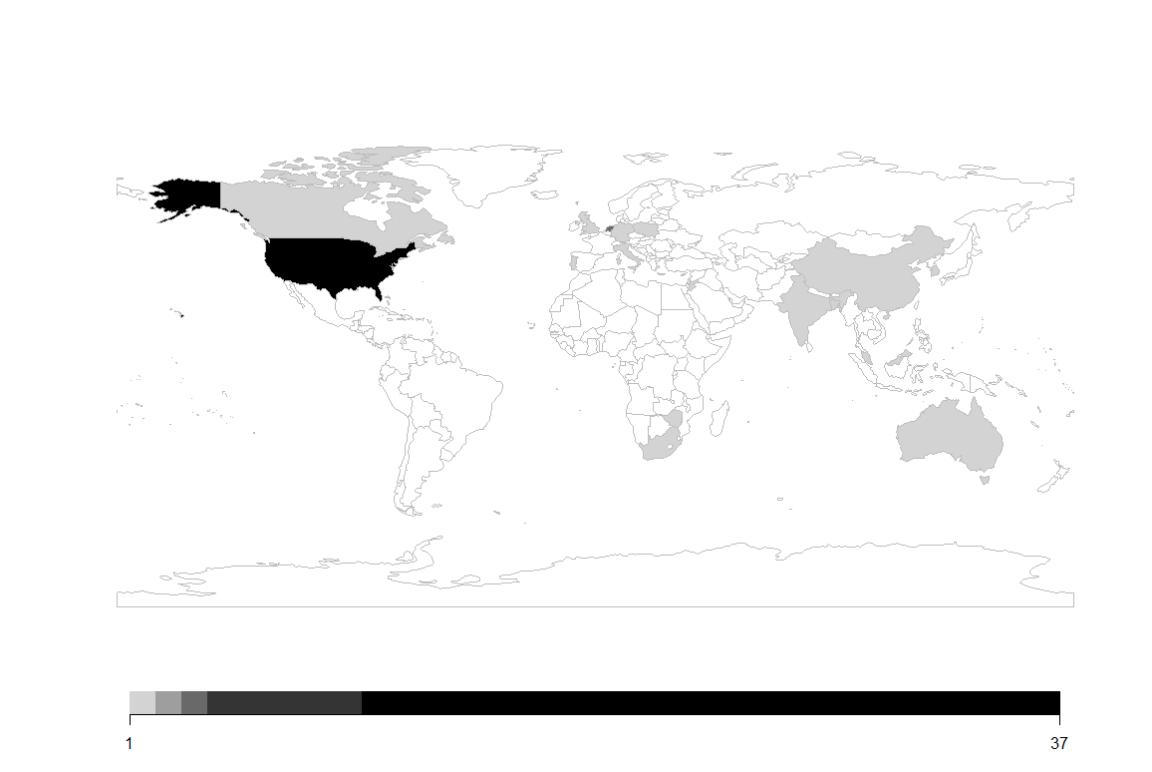
Distribution of the included studies across the globe.

Of the four selected databases, which are PubMed, Web of Science, MEDLINE, and ProQuest, 1990 studies were retrieved, 1170 of which remained after duplicates were removed. Screening of titles and abstracts left us with 143 studies that were subjected to full-text review, yielding a moderate interrater agreement between two researchers (SY and MJC; Cohen κ=0.68) [38]. A total of 62 studies were included in the review (Figure 1) following the PRISMA guidelines (Checklist 1). Additionally, 30 studies were included in the generic inverse variance meta-analysis using the restricted maximum likelihood method. The distribution of included studies is depicted on a world map in Figure 2, and the characteristics of the studies are summarized in Multimedia Appendix 2. The detailed characteristics of all included studies and the inequality indicators listed in the studies are present in Multimedia Appendix 3 [25,39-99].

### Quality Assessment

All 62 studies were subjected to quality assessment according to the Mixed Methods Appraisal Tool (Table 2). Kendall coefficient of concordance was 0.85, indicating very good agreement between the raters (SY and MJC) [100]. Among the selected studies, 90% (n=56) were of high quality. Randomized controlled trials (RCTs) had lower ratings, especially for information regarding outcome assessor blinding to the intervention, with only 1 of 5 studies provided. Most included studies were observational studies, all of which met three criteria, including exposure or outcome measurement, complete outcome data, and intervention (or exposure), as intended. However, some of the included studies did not provide sufficient information on representative populations or adjustment for confounders.

**Table 2.** Quality assessment summary of included studies.

Criteria for quality assessment	Meeting criteria, n (%)
Qualitative (n=10)	
Appropriate answer to the research question	10 (100)
Adequate data collection	10 (100)
Adequate findings from the data	10 (100)
Verified interpretation	9 (90)
Coherence	10 (100)
Randomized controlled trials (n=5)	
Appropriate randomization	4 (80)
Comparable groups at baseline	2 (40)
Completion of outcome data	5 (100)
Blinding of assessors	1 (20)
Adherence to the intervention	4 (80)
Nonrandomized (observational; n=47)	
Representative population	38 (81)
Exposure or outcome measurement	47 (100)
Completion of outcome data	47 (100)
Adjustment of confounders	37 (79)
Intervention or exposure as intended	47 (100)

### Inequality Indicators by Phase

In 14 (23%) studies, results from multiple phases were seen in a single study [25,39-51]. Of the studies considered, 23 (37%) studies were included in the access phase. The outcome variables for the access phase encompass having mHealth apps for health-seeking behavior [25,39-42,44,47,48,52,53], owning a smartphone, digital devices, or mobile phone [41,45,46,48,49,51,52,54-59], and access to mHealth, including fitness trackers [60]. Additionally, proficiency in using mHealth [50] or the need for assistance using mHealth was considered as the outcome for access to mHealth [61].

In total, 47 (76%) studies covered the adoption of mHealth. One example is the number of individuals who signed up for the health program delivered through the website and mobile app each week (weekly subscription rate) [62], or just the adoption of mHealth in the specific population [43,63-67]. Most studies used the use of mHealth apps and digital health tools as an indicator of mHealth adoption [32,39,41,44,46,48,49,68-79]. Additionally, downloads of health apps from some studies were considered a proxy for the adoption of mHealth; the decision to download mHealth apps can be seen as an indication of the acceptance of mHealth to a reasonable degree [80,81]. Furthermore, two studies incorporated outcomes related to the use of wearables [82,83]. Other studies investigated the adoption of mHealth with mobile phone utilization [45,84], behavioral intention to use mHealth [85-87], willingness to use [47,88-90], engagement with a mobile app [77,91,92], attitude toward mHealth or technology [50,93,94], perceived usability [53], and acceptability and cultural relevance of a culturally adapted mHealth [95]. Another study showed differences in the blood glucose levels achieved at the adoption level [96].

In total, 9 (15%) studies were related to mHealth adherence. The studies included in this phase had outcome variables, such as engagement with mHealth or mobile interventions [42,43,77,91,92,97], and continuing to use the app [39,44]. Another study demonstrated app adherence and quit attempts among smokers after preparation [98].

**Figure 3.**
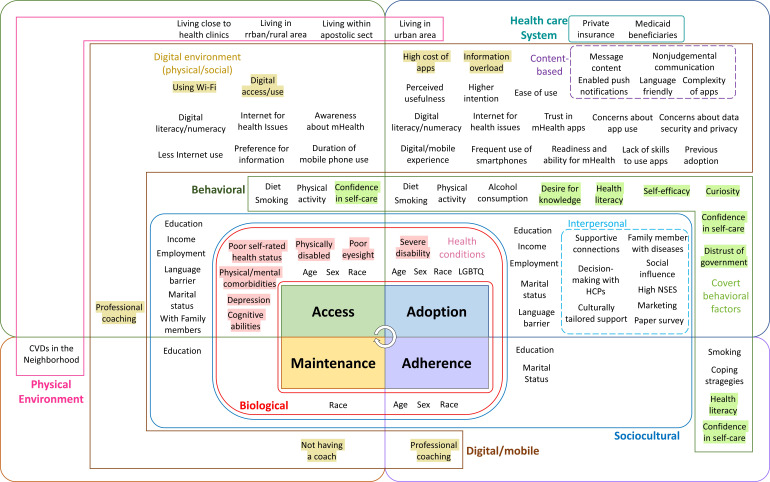
Framework for mHealth inequality indicators based on domains of influence across the implementation phases. CVD: cardiovascular disease; HCP: health care provider. mHealth: mobile health; NSES: neighborhood socioeconomic status.

Only 2 (3%) studies considered the maintenance of mHealth use, while an RCT examined the effectiveness of a 60-day SMS text message intervention for depression and anxiety symptoms; the latter research was based on the RE-AIM (Reach, Effectiveness, Adoption, Implementation, and Maintenance) framework [42]. Another study identified factors leading to nonuse attrition in an RCT involving a technology-based intervention aimed at enhancing self-management behaviors among Black adults at heightened risk of cardiovascular conditions over 6 months [99]. After organizing all the inequality indicators of mHealth use, a visual framework representing the extracted factors by phase was developed, as shown in Figure 3. All the specific factors are listed by phase and levels of influence (Multimedia Appendix 4) [25,39-99].

### Meta-Analysis

**Figure 4.**
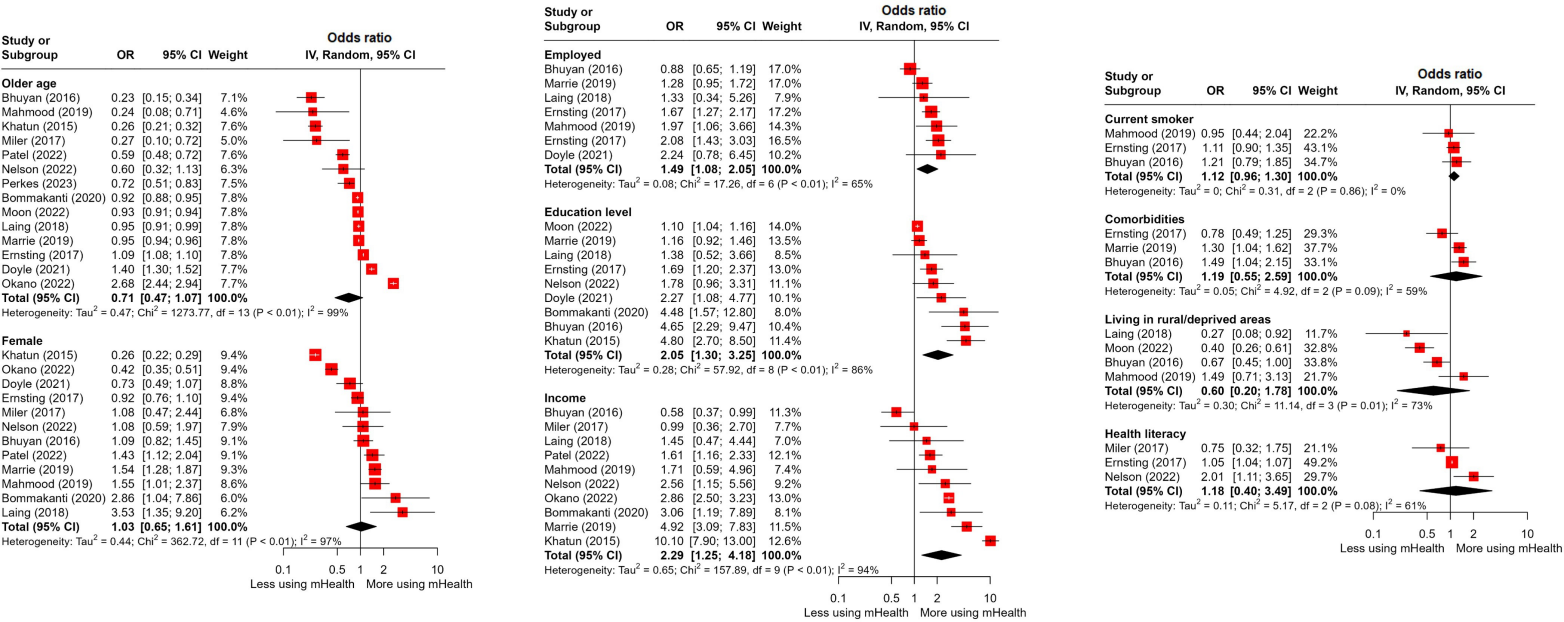
Forest plot displaying the synthesized effect sizes of mHealth utilization based on inequality indicators during the access phase [25,39-41,46-49,52,54-61,66,75,76,81,87-90]. mHealth: mobile health; OR: odds ratio.

**Figure 5.**
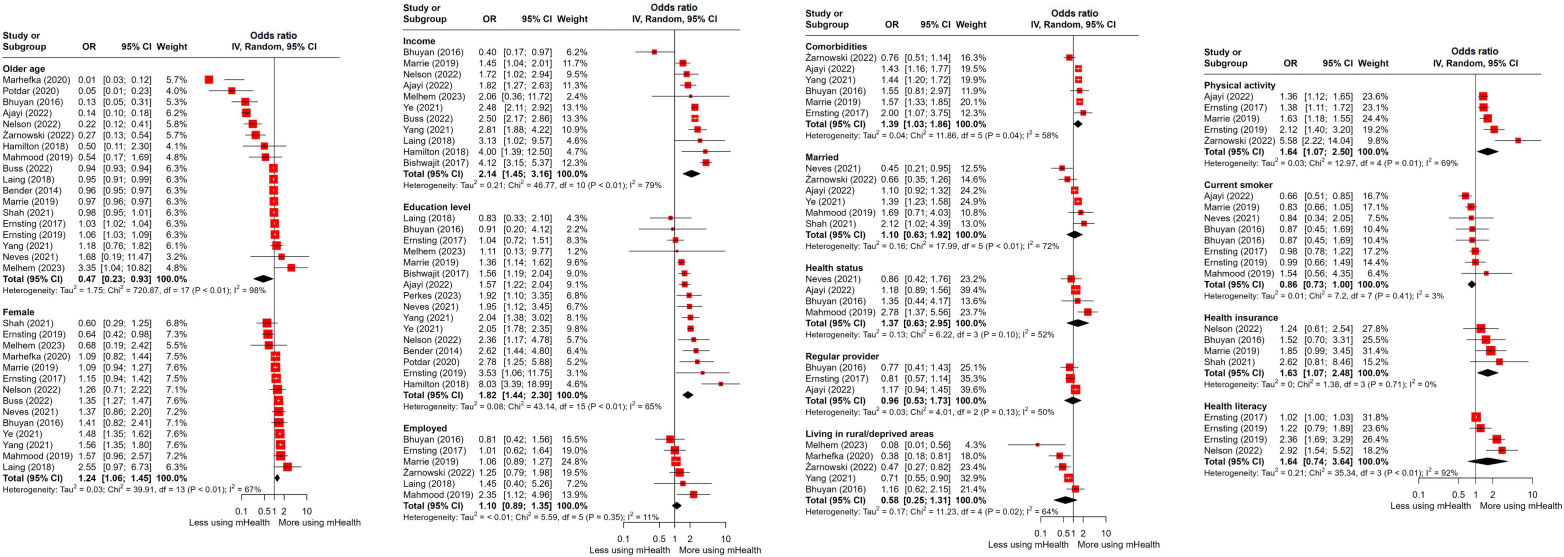
Forest plot displaying the synthesized effect sizes of mHealth utilization based on inequality indicators during the adoption phase [25,39,41,46,48,49,54,57,59,61,66,69,75,76,80,81,83,84,87-90,98,99]. mHealth: mobile health; OR: odds ratio.

Among the implementation phases of mHealth utilization, meta-analyses were available only for the access (Figure 4) and adoption phases (Figure 5) according to the inclusion criteria, which required 3 or more studies for each inequality indicator. When an inequality indicator was dichotomous and had different directions of study effects, the value in one direction was inversely estimated to match that of the other. As a result of meta-analyses, older age (OR 0.47, 95% CI 0.23-0.93) had a significantly negative association with mHealth utilization in the adoption phase. Conversely, a higher education level was positively related to mHealth use in both the access (OR 2.05, 95% CI 1.30-3.25) and adoption phases (OR 1.82, 95% CI 1.44-2.30), and these were statistically significant. Likewise, higher income was positively associated with the use of mHealth in both the access (OR 2.29, 95% CI 1.25-4.18) and adoption phases (OR 2.14, 95% CI 1.45-3.16), with statistical significance. Employment status was positively associated with mHealth utilization, but it was statistically significant only in the access phase (OR 1.49, 95% CI 1.08-2.05). Furthermore, having more comorbidities (OR 1.39, 95% CI 1.03-1.86) and having (private over public) health insurance (OR 1.63, 95% CI 1.07-2.48) were statistically significant for the association with mHealth use in the adoption phase. Despite being statistically insignificant, health literacy was positively associated with mHealth utilization in both the access and adoption phases, unlike living in rural or deprived areas. Current smokers were more inclined to access mHealth services, but their likelihood of adopting them was lower, though this difference was not statistically significant. Female (OR 1.24, 95% CI 1.06-1.45) and those prone to physical activity (OR 1.64, 95% CI 1.07-2.50) were more likely to adopt mHealth. Race or ethnicity was not significantly associated with mHealth utilization (Multimedia Appendix 5) [25,39,46,49,59-61,66,69,75,76,80,87,90]. Publication bias was assessed by funnel plots (Multimedia Appendix 6).

## Discussion

### Principal Findings

This study highlights inequalities in mHealth utilization across the phases of access, adoption, adherence, and maintenance through a comprehensive systematic review and meta-analysis. We also provide exhaustive insights into the factors influencing mHealth use in each phase, with the most significant inequalities identified during the access and adoption phases. All the study findings are encapsulated in the conceptual framework proposed in Figure 3, which illustrates how biological, sociocultural, behavioral, environmental, digital or mobile, and health care system factors affect all phases of mHealth utilization. As most studies have focused on the access and adoption phases, it was difficult to investigate the subsequent phases. By embedding this conceptual framework across all phases, we provide a structured approach for understanding and addressing the inequalities in mHealth engagement, underscoring the importance of targeting interventions to specific phases while also recognizing the interconnectedness of the domains involved.

Biological factors, such as age, gender, and health conditions, affect mHealth use in terms of access, adoption, and adherence. Age stood out as a key factor, with younger people using mHealth apps the most, as older adults often face difficulties in mobile device ownership and technology adoption [54,101]. Comorbidities influence access to mHealth utilization, possibly owing to a greater need to manage multiple health conditions. This finding reflects the concerns raised by previous research regarding the usability and accessibility of mHealth tools for older individuals with multiple health conditions [102]. Race was not consistently linked to mHealth use across all phases, with the insignificance of the meta-analysis.

This study showed gender differences in that women were more likely to adopt mHealth services than men. This aligns with previous research, suggesting that women generally engage more in health-related activities and are more proactive in their health management [103]. Conversely, men may exhibit lower engagement due to factors such as lower health consciousness or different health-seeking behaviors. Recognizing these gender differences is crucial for developing targeted strategies to promote mHealth utilization among men, possibly through awareness campaigns or by designing apps that cater to their specific health interests and needs.

Education level was consistently associated with mHealth utilization throughout every phase. Our meta-analysis highlighted that individuals with higher education and income have more than double the odds of accessing mHealth compared to those with lower education and income, indicating the need for targeted interventions to improve digital infrastructure and literacy among disadvantaged groups. Education and digital literacy continue to play pivotal roles, as individuals with higher education levels and digital familiarity tend to be better equipped to adopt mHealth solutions. This could be attributed to better health literacy and greater familiarity with digital tools among more educated individuals [104].

Behavioral factors, both covert (eg, motivation) and overt (eg, health behaviors), become increasingly important as users progress from adoption to adherence. It was also confirmed that users who are proactive about their health—those engaged in regular physical activity—are more likely to adopt the mHealth tool [105]. Personal motivation, health literacy, and sustained engagement with health behaviors remain central to continued use of mHealth tools. Although we cannot confirm how these factors interact, behavioral motivation and sustained engagement in health literacy efforts may play key roles in ensuring adherence among older adults and other disadvantaged populations.

Environmental factors, such as access to health care infrastructure and geographic location, predominantly impact the access phase; however, improvements in digital infrastructure can also enhance both adoption and maintenance. Individuals in rural or underserved areas often encounter challenges with internet access, limiting their ability to use mHealth technologies [106]. However, further longitudinal studies are needed to explore the role of behavioral and environmental factors in long-term engagement.

Digital and mobile factors, including the ongoing availability of support and clear communication, are important for users when it comes to remaining engaged. The adoption phase is strongly influenced by digital or mobile factors such as familiarity with technology. The usability and perceived usefulness of a platform, along with trust in technology, are central to whether individuals adopt these solutions. Digital literacy also plays a crucial role, as those with lower digital skills are less likely to access mHealth solutions. Therefore, building digital capacity in the general population should be a key goal to ensure that everyone can optimally, equitably, and sustainably benefit from advancements in the digital era [107]. Regarding content-based factors, developing motivational SMS text messages using a user-centered design could be beneficial for low-income populations with low health literacy and those with language barriers [108]. Therefore, a reflection on research concerning content analysis and quality assessment of mHealth apps, which has often been neglected, emphasizes the significance of usability and functionality in app development [109]. This may help mitigate inequalities stemming from content-based factors in mHealth utilization among end users [110-113].

Health care system factors are also crucial throughout the journey of using mHealth, ensuring that users remain engaged over time. The integration of mHealth into routine care and support from health care providers, and having proper health insurance, significantly influence adoption. External support from family members is also important in maintaining engagement, especially among older adults and those with lower digital literacy. As mHealth, including digital therapeutics, is poised to transform health care delivery by challenging the core assumption that health care must be location-specific and episodic [114], a multistakeholder approach can be considered to provide a useful means by which policy makers can assess their health system’s readiness for mHealth [115].

In summary, digital tools often neglect the specific needs of vulnerable populations, hindering their access to essential health services and worsening health inequalities [107]. Older adults, people in rural areas, and those with disabilities face the highest risk of digital exclusion [116]. While digital technology has great potential, policy and global digital literacy must keep pace with technological progress [117]. Reflecting on these facts, it is important that mHealth be available to everyone, not just affluent populations. Hence, policies should address concerns about reimbursement, safety, and privacy. This indicates the need for additional regulatory progress in areas such as operationalization, implementation, and the transferability of international approvals. Collaborative regulatory efforts across countries are vital to fully leverage the potential of these technologies [109]. Future studies are warranted to better understand the policy- and regulation-related factors affecting mHealth utilization. Furthermore, because mHealth apps are distributed through diverse channels, strategies for marketing mHealth apps for regular use in the health care sector should be investigated [118].

### Limitations

This study has some limitations. First, more related studies, non-English studies, and gray literature may exist but were excluded due to the focus on mHealth within four databases and language barriers, which might limit the generalizability. Nevertheless, this issue is likely minor, as we used a highly sensitive search strategy aimed at capturing as many relevant studies as possible. In addition, despite using a literature review tool for a systematic and efficient title and abstract review, the single-author process may have introduced bias by missing relevant papers. The results of the meta-analysis could be overestimated or underrepresented without considering excluded studies, such as gray literature, or literature using measures other than the OR. Furthermore, the directionality and causality of the factors identified in this study cannot be conclusively established, as this review mainly relies on cross-sectional or retrospective studies. Finally, although ORs in meta-analysis may raise concerns about heterogeneity, we addressed this by using the *I²* statistic and a restricted random-effects model to minimize its impact.

The lack of sufficient data on the adherence and maintenance phases also presents a research gap, particularly in understanding how users sustain long-term engagement with mHealth technology. Future research may need to focus on using sophisticated longitudinal study designs that allow for causal inference and a deeper exploration of how factors evolve over time and interact across all phases of mHealth utilization. Additionally, expanding the scope to include a more diverse range of populations and geographic regions will help address the global inequalities in mHealth access, adoption, and utilization. This could offer valuable insights into how cultural, social, and economic contexts shape mHealth engagement. However, as mHealth technologies continue to evolve rapidly, the findings of this study may not be fully generalizable to emerging platforms such as virtual reality. Furthermore, given the diverse populations and regional characteristics across different parts of the world, it would be valuable to conduct in-depth research examining how these characteristics vary and the factors associated with them in each region.

### Conclusions

In conclusion, while identifying the factors influencing mHealth utilization does not fully explain health inequalities solely attributable to mHealth use, these associations may significantly impact health outcomes and contribute to inequalities. The conceptual framework outlined in this study highlights the multifaceted nature of mHealth utilization across all the phases of mHealth engagement: access, adoption, adherence, and maintenance. To address these inequalities, tailored and personalized interventions are required at each phase of mHealth utilization. Targeted efforts can enhance digital access for older and low-income adults while promoting engagement through education, insurance support, and healthy behaviors, thereby promoting equitable and effective mHealth use. By recognizing the interconnectedness of these domains, policy makers and health care stakeholders can design interventions that not only address the phase-specific barriers but also bridge broader inequalities in health care access and engagement through research on each relevant factor in the region where this is to be applied.

## Supplementary material

10.2196/71349 Multimedia Appendix 1 Literature search strategy and keywords.

10.2196/71349 Multimedia Appendix 2 Characteristics of included studies by category (n=62).

10.2196/71349 Multimedia Appendix 3 Overview of included studies.

10.2196/71349 Multimedia Appendix 4 All factors associated with mHealth inequalities by phase and measures of the association.

10.2196/71349 Multimedia Appendix 5 Forest plot showing synthesized effect sizes of mHealth utilization by race/ethnicity in the access (a) and adoption (b) phase.

10.2196/71349 Multimedia Appendix 6 Funnel plot of included studies for meta-analysis showing factors in the access (a) and adoption (b) phase.

10.2196/71349 Checklist 1 PRISMA Checklist

## Abbreviations

mHealth: mobile health
OR: odds ratio
PRISMA: Preferred Reporting Items for Systematic Reviews and Meta-Analyses
RCT: randomized controlled trial
RE-AIM: Reach, Effectiveness, Adoption, Implementation, and Maintenance
