# Supplementary material for: Understanding Inequalities in Mobile Health Utilization Across Phases: Systematic Review and Meta-Analysis
Source: J Med Internet Res. 2025 Aug 14;27:e71349. doi: 10.2196/71349 (PMC12352709; doi:10.2196/71349)
Supplement: Multimedia Appendix 1 [file jmir-v27-e71349-s001.docx]

| Search strategy for PubMed | Search strategy for Medline |
| --- | --- |
| 1. (((((digital therapeutic*[Title/Abstract]) OR (DTx[Title/Abstract])) OR (mHealth[Title/Abstract])) OR (mHealth app*[Title/Abstract])) OR (mobile intervention*[Title/Abstract])) OR (mobile health intervention*[Title/Abstract])  2. ((digital divide[MeSH Terms]) OR (health status disparities[MeSH Terms])) OR (healthcare disparities[MeSH Terms])  3. ((((((((digital divide[Title/Abstract]) OR (health status disparit*[Title/Abstract])) OR (inequalit*[Title/Abstract])) OR (inequit*[Title/Abstract])) OR (disparit*[Title/Abstract])) OR (digital inequalit*[Title/Abstract])) OR (digital inequit*[Title/Abstract])) OR (digital equalit*[Title/Abstract])) OR (digital equit*[Title/Abstract])  4. #1 AND (#2 OR #3) | 1. TI digital therapeutic* OR TI DTx OR TI mhealth OR TI mhealth app* OR TI digital intervention* OR TI mobile intervention* OR TI digital health intervention* OR TI mobile health intervention*  2. AB digital therapeutic* OR AB DTx OR AB mhealth OR AB mhealth app* OR AB digital intervention* OR AB mobile intervention* OR AB digital health intervention* OR AB mobile health intervention*  3. TI digital divide OR TI health status disparit* OR TI inequalit* OR TI inequit* OR TI disparit* OR TI digital inequalit* OR TI digital inequit* OR TI digital equalit* OR TI digital equit*  4. AB digital divide OR AB health status disparit* OR AB inequalit* OR AB inequit* OR AB disparit* OR AB digital inequalit* OR AB digital inequit* OR AB digital equalit* OR AB digital equit*  5. (S1 OR S2) AND (S3 OR S4) |
| Search strategy for Web of Science | Search strategy for ProQuest |
| 1. ((((((TI=(digital therapeutic*)) OR TI=(DTx)) OR TI=(mHealth)) OR TI=(mHealth app*)) OR TI=(mobile intervention*)) OR TI=(mobile health intervention*))  2. ((((((AB=(digital therapeutic*)) OR AB=(DTx)) OR AB=(mHealth)) OR AB=(mHealth app*)) OR AB=(mobile intervention*)) OR AB=(mobile health intervention*))  3. ((((((((TI=(digital divide)) OR TI=(health status disparit*)) OR TI=(inequalit*)) OR TI=(inequit*)) OR TI=(disparit*)) OR TI=(digital inequalit*)) OR TI=(digital inequit*)) OR TI=(digital equalit*)) OR TI=(digital equit*)  4. ((((((((AB=(digital divide)) OR AB=(health status disparit*)) OR AB=(inequalit*)) OR AB=(inequit*)) OR AB=(disparit*)) OR AB=(digital inequalit*)) OR AB=(digital inequit*)) OR AB=(digital equalit*)) OR AB=(digital equit*)  5. (#1 OR #2) AND (#3 OR #4) | ((title(digital therapeutic*) OR title(DTx) OR title(mHealth) OR title(mobile intervention*)) OR (abstract(digital therapeutic*) OR abstract(DTx) OR abstract(mHealth) OR abstract(mobile intervention*))) AND ((title(digital divide) OR title(health status disparit*) OR title(inequalit*) OR title(inequit*) OR title(disparit*) OR title(digital inequalit*) OR title(digital inequit*)) OR (abstract(digital divide) OR abstract(health status disparit*) OR abstract(inequalit*) OR abstract(inequit*) OR abstract(disparit*) OR abstract(digital inequalit*) OR abstract(digital inequit*))) |
