# Supplementary material for: Understanding Inequalities in Mobile Health Utilization Across Phases: Systematic Review and Meta-Analysis
Source: J Med Internet Res. 2025 Aug 14;27:e71349. doi: 10.2196/71349 (PMC12352709; doi:10.2196/71349)
Supplement: Multimedia Appendix 2 [file jmir-v27-e71349-s002.docx]

| Category | | Count (%) |
| --- | --- | --- |
| All | | 62 (100) |
| Region | | |
| Africa | | 4 (6.5) |
|  | South Africa | 2 (3.2) |
|  | Seychelles | 1 (1.6) |
|  | Zimbabwe | 1 (1.6) |
| Asia/Pacific | | 8 (12.9) |
|  | Australia | 2 (3.2) |
|  | Bangladesh | 2 (3.2) |
|  | China | 1 (1.6) |
|  | India | 1 (1.6) |
|  | Malaysia | 1 (1.6) |
|  | South Korea | 1 (1.6) |
| Europe | | 11 (17.7) |
|  | Germany | 2 (3.2) |
|  | Italy | 1 (1.6) |
|  | Netherlands | 4 (6.5) |
|  | Poland | 1 (1.6) |
|  | Portugal | 1 (1.6) |
|  | United Kingdom | 2 (3.2) |
| Middle East | | 1 (1.6) |
|  | Jordan | 1 (1.6) |
| North America | | 38 (61.3) |
|  | Canada | 1 (1.6) |
|  | United States | 37 (59.7) |
| Study type | | |
|  | Cross-sectional | 23 (37.1) |
|  | Prospective | 8 (12.9) |
|  | Qualitative | 10 (16.1) |
|  | Randomized controlled trial | 5 (8.1) |
|  | Retrospective | 16 (25.8) |
