# Supplementary material for: Understanding Inequalities in Mobile Health Utilization Across Phases: Systematic Review and Meta-Analysis
Source: J Med Internet Res. 2025 Aug 14;27:e71349. doi: 10.2196/71349 (PMC12352709; doi:10.2196/71349)
Supplement: Multimedia Appendix 3 [file jmir-v27-e71349-s003.docx]

| No. | Ref. | First author | Year | Settings | Study type | Target outcome | Population | Health condition | Sample size | Mean age | Type of intervention | Mode of delivery |
| --- | --- | --- | --- | --- | --- | --- | --- | --- | --- | --- | --- | --- |
| 1 | 62 | Agachi | 2022 | Netherlands | Prospective | Adoption of a mobile app | SamenGezond platform, the general Dutch population | Prevention | 83466 | 46.5 | A web-based Preventive Health Program | Smartphones and website |
| 2 | 68 | Ajayi | 2022 | USA | Retrospective | Use of digital health tools for health promotion  (Tablet to achieve goals) | Women from the Health Information Trends Survey (HINTS) Cycle 1-4 | Chronic diseases | 8564 | N/A | N/A | Smartphones or tablets |
| 3 | 80 | Bender | 2014 | USA | Cross-sectional | Health app downloads | Mobile phone-based diabetes prevention program for at-risk Filipinos, Koreans, and Latinos | No history of diabetes | 904 | 44 | N/A | Mobile phones |
| 4 | 39 | Bhuyan | 2016 | USA | Retrospective | Having and using of Mobile Health Applications for Health-Seeking Behavior  (Access: Having  mHealth apps/ Adoption: Use of mHealth  apps for achieving health  behavior goals) | Health Information National Trends Survey (HINTS 4) cycle 4 | Behavior change | 3677 | N/A | N/A | Smartphones or tablets |
| 5 | 84 | Bishwajit | 2017 | Bangladesh | Cross-sectional | Mobile phone utilization status for seeking childbirth services | Married women aged between 15 and 49 years from Bangladesh Urban Health Survey (BUHS) 2013 | Childbirth | 9014 | 25.9 | N/A | Mobile phones |
| 6 | 54 | Bommakanti | 2020 | USA | Cross-sectional | Not owning a smartphone | People receiving treatment for confirmed or suspected TB | Tuberculosis | 151 | 41 | Video Directly Observed Therapy | Smartphones |
| 7 | 40 | Bonnell | 2022 | USA | Qualitative | Access and uptake inequalities of WA Notify as the community-level adoption | Community leaders and representative of the diversity of populations and regions across WA state | N/A | 17 | N/A | N/A | Smartphones |
| 8 | 69 | Buss | 2022 | Australia | Cross-sectional | Mobile health app use | 45 and Up Study from the Services Australia Medicare enrollment database | Risk of cardiovascular disease and type 2 diabetes mellitus | 31946 | 69 (median) | N/A | Mobile phones or tablets |
| 9 | 70 | Camacho-Rivera | 2020 | USA | Retrospective | COVID-19 mHealth tool use | COVID-19 Household Impact Survey | Chronic health conditions | 10760 | N/A | N/A | Mobile health tools |
| 10 | 71 | Cao | 2022 | China | Cross-sectional | mHealth use | 12 tertiary hospitals across three cities of the Inner Mongolia Autonomous Region | Patients and caregivers | 2115 | 43.3 | N/A | Smartphones |
| 11 | 72 | Che Johan | 2023 | Malaysia | Cross-sectional | Use of mHealth applications | Hospital Universiti Sains Malaysia | Chronic kidney disease | 100 | 62.1 | N/A | Smartphones or tablets |
| 12 | 73 | Chen | 2023 | USA | Retrospective | mHealth usage | Health Information National Trends Survey 5, (HINTS 5), Aged >=18 | Hypertension | 4893 | 61 | N/A | Smartphones |
| 13 | 85 | Choudhury | 2023 | India | Cross-sectional | Behavioral intention to use mobile health apps | Pregnant mothers aged 15-49 in three underserved villages in Jharkhand, India | Anemia and preeclampsia | 131 | N/A | N/A | Smartphones |
| 14 | 86 | Cilliers | 2017 | South Africa | Cross-sectional | Intention to use mobile devices to search for health-related queries | University students at two campuses of a traditional university in the Easern Cape, South Africa | N/A | 202 | N/A | N/A | Mobile phones |
| 15 | 55 | Doyle | 2021 | Zimbabwe | Cross-sectional | Mobile phone ownership | Aged 13-24 years in 5 communities in urban and peri-urban Harare and Mashonaland East, Zimbabwe. | N/A | 634 | 18 | N/A | Mobile phones |
| 16 | 41 | Ernsting | 2017 | Germany | Cross-sectional | Smartphone and health app use | A population-based sample from Germany | Behavior change | 4144 | 57 | N/A | Smartphones |
| 17 | 81 | Ernsting | 2019 | Germany | Cross-sectional | Mobile health app download | A population-based sample from Germany | Cardiovascuar disease or diabetes | 1500 | 55.1 | N/A | Smartphones or tablets |
| 18 | 74 | Fradkin | 2022 | USA | Retrospective | Use of a free tobacco cessation app | WA residents who registered for and activated the 2Morrow Health Smoking & Tobacco app | Tobacco Cessation | 1280 | N/A | Self-guided smartphone app | Smartphones |
| 19 | 96 | Gershoni | 2023 | USA | Retrospective | Differences in the blood glucose levels achieved by several racial/ethnic groups using the same digital tool | DarioTM database on individuals who used the Dario platform between 2019–2021 | Hyperglycemia, high-risk | 1000 | N/A | Smartphone app | Smartphones |
| 20 | 82 | Ginossar | 2021 | USA | Qualitative | Wearable activity trackers (WATs) adoption | Rural older cancer survivors who live in New Mexico | Cancer survivors | 31 | N/A | N/A | Wearables |
| 21 | 75 | Hamilton | 2018 | USA | Cross-sectional | Level of mHealth technology utilization | Caregivers of pediatric surgery patients | N/A | 171 | 36 | N/A | Smartphones |
| 22 | 91 | Hardy | 2022 | UK | Randomized controlled tral | Mobile app engagement in the SlowMo | Age of ≥18 years; persistent (≥3 months) distressing paranoia; a diagnosis of schizophrenia-spectrum psychosis | Phychosis | 168 | N/A | SlowMo mobile app | Smartphones |
| 23 | 42 | Haro-Ramos | 2023 | USA | Randomized controlled tral | Effectiveness of StayWell (text message intervention) | Latinx and Non-Latinx White users | Depression and anxiety symptoms | 398 | 37.5 | Text messaging | Mobile phones |
| 24 | 43 | Hengst | 2023 | Netherlands | Prospective | Adoption and indications of public health apps | Respondents from the Longitudinal Internet Studies for the Social Sciences (LISS) panel at Tilburg University | N/A | 1900 | 51.8 | CoronaMelder, contact-tracing apps | Smartphones |
| 25 | 99 | Idris | 2022 | USA | Prospective | Nonusage attrition (usage logs of time-stamped events) | Black adults with poor cardiovascular health was taken from a clinical intervention | Multiple cardiovascular risk factors | 132 | 55.2 | Technology-based behavioral intervention | Smartphones |
| 26 | 44 | Jiwani | 2023 | USA | Retrospective | Accessing and continuing to utilize the app | HMP users who had downloaded the app from a US Apple or Google app store | N/A | 66482 | N/A | Healthy Minds Program (HMP), a meditation-based well-being app | Smartphones |
| 27 | 52 | Khatun | 2015 | Bangladesh | Cross-sectional | Readiness for mHealth (Ownership, knowledge) | Residents in Chakaria sub-district | N/A | 4915 | N/A | N/A | Mobile phones |
| 28 | 45 | Kim | 2015 | USA | Qualitative | Smartphone use for health information | Low-SES adults self-identified as Hispanics | N/A | 20 | N/A | N/A | Smartphones |
| 29 | 76 | Kim | 2021 | South Korea | Prospective | Health app use (frequency of health app use and the number of types) | Two-wave opt-in panel survey of Korean adults | N/A | 440 | 40.4 | N/A | Smartphones or tablets |
| 30 | 87 | Klaver | 2021 | Netherlands | Cross-sectional | Intention to use mHealth | Older adults over the age of 65 years in the Netherlands | N/A | 463 | N/A | N/A | Smartphones or tablets |
| 31 | 46 | Laing | 2018 | USA | Cross-sectional | Knowledge – awareness and practice of using smartphone for wellness (or medical-based apps) | Adults in possession of a smartphone from 5 community health center sites in Washington State and Washington, DC | N/A | 159 | 35.2 | N/A | Smartphones |
| 32 | 63 | Leziak | 2021 | USA | Qualitative | Adoption of evidence-based mHealth technology | Low-income pregnant women with DM | Diabetes | 46 | 31 | Smartphone app | Smartphones |
| 33 | 47 | Luo | 2021 | USA | Qualitative | Willing to try at least one diabetes-related app and barriers to app use | Personal interviewees | Diabetes | 15 | N/A | N/A | Smartphones |
| 34 | 95 | Maglalang | 2017 | USA | Qualitative | Acceptability and cultural relevance of a culturally adapted mHealth weight-loss lifestyle intervention | Filipino Americans in the PilAm Go4Health pilot RCT weight-loss lifestyle interven- tion | Overweight | 45 | N/A | Culturally adapted mobile health weight-loss lifestyle intervention | Smartphones |
| 35 | 25 | Mahmood | 2019 | USA | Retrospective | Use of mobile health applications for health-promoting behavior | Health Information National Trends Survey (HINTS 5, Cycle 1, 2017) | Chronic medical conditions | 1864 | N/A | N/A | Smartphones |
| 36 | 88 | Marhefka | 2020 | USA | Prospective | Willingness to use technology for HIV-related information | HIV/AIDS service organizations providing Ryan White case management | HIV/AIDS | 1268 | 48 | N/A | Mobile phones or tablets |
| 37 | 48 | Marrie | 2019 | USA | Cross-sectional | Use of mobile devices and adoption of mHealth apps | The North American Research Committee on Multiple Sclerosis (NARCOMS) Registry | Multiple sclerosis | 7064 | 60.4 | N/A | Smartphones or tablets |
| 38 | 98 | Meijer | 2021 | Netherlands | Cross-sectional | App adherence to StopCoach | Lower-SES smokers based on StopAdvisor, in a real-world setting | Smoking | 22 | N/A | “De StopCoach”, a mobile phone delivered eHealth intervention app | Smartphones |
| 39 | 89 | Melhem | 2023 | Jordan | Cross-sectional | Willingness to mHealth engagement | All Jordanian breast and colorectal cancer patients of both sexes registered by Jordan’s national cancer registry | Breast and colorectal cancer survivors | 335 | N/A | Self-management | Smartphones |
| 40 | 61 | Miller | 2017 | USA | Cross-sectional | Needing assistance to complete the mPATH mHealth program | People from a randomized controlled trial of an iPad program designed to promote colorectal cancer (CRC) screening | CRC screening | 450 | 57 | mPATH mHealth program | Tablets |
| 41 | 56 | Moon | 2022 | UK | Retrospective | Access to mobile devices (smartphone or tablet) | Women from outpatient breast clinics across England and Wales, and through social media | Breast cancer | 2009 | N/A | N/A | Smartphones or tablets |
| 42 | 92 | Nelson | 2016 | USA | Prospective (Pilot) | Engagement with the mHealth intervention (text messages and Interactive Voice Response) | Low-income adults with type 2 diabetes in Nashville, TN | Type 2 diabetes | 80 | 50.1 | Medication adherence promotion intervention | Smartphones |
| 43 | 77 | Nelson | 2020 | USA | Randomized controlled tral | User engagement in a text message-delivered intervention | Diagnosed with T2D, prescribed a daily diabetes medication | Type 2 diabetes | 248 | 55.8 | Self-care promotion text messages (12mos) | Mobile phones |
| 44 | 49 | Nelson | 2022 | USA | Retrospective | Smartphone ownership and health app use | 2 randomized controlled trials (RCTs) evaluating effects of mobile phonedelivered interventions for diabetes self-management | Type 2 diabetes | 422/330 | 56.3/56.8 | N/A | Smartphones |
| 45 | 78 | Neves | 2021 | Portugal | Retrospective | Use of general health and fitness apps | Adolescents and adults with persistent asthma was recruited for the INSPIRERS studies | Asthma | 514 | N/A | N/A | Smartphones |
| 46 | 57 | Okano | 2022 | South Africa | Retrospective | Ownership of mobile phones | Adults in 33 African countries | N/A | 44224 | N/A | N/A | Mobile phones or smartphones |
| 47 | 60 | Patel | 2022 | USA | Retrospective | Mobile health access (fitness tracker ownership) | Adults aged 45 to 84 years and free of known CVD at baseline | Cardiovascular disease | 2796 | 76.6 | N/A | Wearables (fitness trackers) |
| 48 | 58 | Perkes | 2023 | Australia | Cross-sectional | Ownership of digital devices, current mHealth use, and interest and preferences for future mHealth | Aboriginal and Torres Strait Islander women of reproductive age (16-49 years) | N/A | 379 | 31 | N/A | Smartphones or tablets |
| 49 | 53 | Petros | 2022 | Italy | Randomized controlled tral | Perceived usability of the NEVERMIND | Patients with breast or prostate cancer from the University Hospitals, Turin, Italy, | Breast or prostate cancer | 129 | 58.6 | NEVERMIND (Neurobehavioural Predictive and Personalised Modelling of Depressive Symptoms During Primary Somatic Diseases) | Smartphones |
| 50 | 97 | Pollock | 2023 | USA | Randomized control pilot trial | Improvement of patient activation, engagement, and nutritional behaviors | Patients undergoing their first KT, age 14 to 50 y | Kidney transplant recipients | 16 | 38.4 | mHealth app intervention that provides self-management monitoring and coaching | Smartphones |
| 51 | 90 | Potdar | 2020 | USA | Cross-sectional | Willingness to utilize a daily mobile application for health monitoring | Cancer patients attending the outpatient clinic and infusion center at an academic medical center in Philadelphia | Cancer | 151 | 62.3 | N/A | Smartphones |
| 52 | 64 | Ramaswamy | 2023 | USA | Mixed-methods (Qualitative) | Adoption of mHealth apps and usefulness of app features | Aged over 18 years with any stroke (ischemic or hemorrhagic), and ability to communicate | Stroke | 1194 | 62.3 | mHealth app | Smartphones |
| 53 | 93 | Schoenberg | 2021 | USA | Qualitative | Perspectives on this mobile health (mHealth) intervention | Rural residents, including those from the central Appalachian region | N/A | 54 | 47.9 | mHealth app or personilized health coaching intervention | Smartphones |
| 54 | 50 | Schrauben | 2021 | USA | Cross-sectional | Attitudes toward technologies, and proficiency in using mHealth | Persons with mild to moderate CKD in the United States | Chronic kidney disease | 932 | 67.9 | N/A | Smartphones |
| 55 | 79 | Shah | 2021 | USA | Prospective | Digital health intervention use | Type 1 acute myocardial infarction patients ages 18 years and older | Readmission after acute myocardial infarction | 133 | 58.3 | Digital health intervention (adhering to guideline-directed medical therapy) | Smartphones |
| 56 | 65 | Steinberg | 2022 | USA | Prospective | mHealth adoption | Low-income pregnant individuals with gestational or type 2 diabetes mellitus | Gestational or type 2 diabetes mellitus | 24 | 32.1 | mHealth application | Smartphones |
| 57 | 94 | Umaefulam | 2022 | Canada | Qualitative | Perception on using mobile health as a tool for receiving health information | Indigenous women with or at risk of diabetes, aged 18–69 years in Saskatoon, Canada | Diabetes | 22 | N/A | mHealth intervention | Mobile phones |
| 58 | 66 | Yang | 2021 | USA | Retrospective | Adoption of health information management and mobile health for self-regulation | Medicaid population from the Health Information National Trends Survey from 2017 to 2019 | N/A | 9481 | N/A | N/A | Smartphones or tablets |
| 59 | 51 | Ye | 2021 | USA | Retrospective | Ownership and usage of different mHealth technologies | Health Information National Trends Survey from 2017 to 2019 | Physical activities | 12227 | 56.8 | N/A | Smartphones or wearables |
| 60 | 59 | Yepes | 2016 | Seychelles | Retrospective | Mobile technology ownership | Adults aged 25-64 years of the 3 main islands of Seychelles | N/A | 1240 | N/A | N/A | Mobile phones or smartphones |
| 61 | 67 | Yu | 2021 | USA | Mixed-methods (Qualitative) | mHealth adoption | First-generation Chinese and Hispanic immigrants diagnosed with T2DM | Type 2 diabetes | 118 | 61 | T2DM self-management app | Smartphones |
| 62 | 83 | Żarnowski | 2022 | Poland | Cross-sectional | Level of use of mobile apps and wearables | Adult inhabitants of Poland | Controlling diet, weight, and physical activity | 1070 | 45.1 | N/A | Smartphones or wearables |
