# Supplementary material for: Understanding Inequalities in Mobile Health Utilization Across Phases: Systematic Review and Meta-Analysis
Source: J Med Internet Res. 2025 Aug 14;27:e71349. doi: 10.2196/71349 (PMC12352709; doi:10.2196/71349)
Supplement: Multimedia Appendix 4 [file jmir-v27-e71349-s004.docx]

| **No.** | **Ref.** | **First author** | **Year** | **Phase** | | | | **Level of influence** | **Type of estimate** | **Estimates** | **Included in Meta-analysis** |
| --- | --- | --- | --- | --- | --- | --- | --- | --- | --- | --- | --- |
|  |  |  |  | Access | Adoption | Adherence | Maintenance |  |  |  |  |
| 1 | 62 | Agachi | 2022 |  | Male (N) Age (N) High NSES (N) Marketing (P) |  |  | Community | Hazard ratios for adoption | Male (N) 0.821 (0.797-0.845) Age (N) 0.980 (0.979-0.981) High NSES (N) 0.948 (0.910-0.987) Marketing (P) 17.007 (16.979-17.035) |  |
| 2 | 70 | Ajayi | 2022 |  | Multiple chronic conditions (P)  Age (N)  Low income (N)  NH h Blacks (P)  College degree or more (P)  Active physical activity (P)  Current smoker (N) Previous smoker (P) |  |  | Individual | Odds ratios for adoption | Multiple chronic conditions (P) aOR 1.43 (1.16-1.77)  Age (N) 0.14 (0.10-0.18)  Low income (N) 0.55 (0.38-0.79)  NH h Blacks (P) 1.44 (1.12-1.85)  Active physical activity (P) 1.36 (1.12-1.65)  Current smoker (N) 0.66 (0.51-0.85)  Previous smoker (P) 1.24 (1.02-1.52)  Married 1.10 (0.92-1.32)  Hispanics 1.09 (0.83-1.44)  Asians/others 1.25 (0.88-1.79)  College degree or more (P) 1.57 (1.22-2.04)  Insurance 1.01 (0.69-1.49)  Poor health status 0.85 (0.64-1.12)  Regular provider 1.17 (0.94-1.45) | X |
| 3 | 80 | Bender | 2014 |  | Age (N) Korean, Latino (N) (vs Caucasian) Higher education level (P) Family member with MI (P) Paper survey (N) |  |  | Individual | Odds ratios for adoption | Age (N) OR 0.96, 95% CI 0.95-0.97 Korean (N) OR 0.52, 95% CI 0.31-0.88 Latino (N) (vs Caucasian) OR 0.37, 95% CI 0.20-0.69  Filipino 0.89 (0.54-1.48) Higher education level (P) college (OR 2.62, 95% CI 1.44-4.80) or graduate school (OR 2.93, 95% CI 1.43-6.00) Family member with MI (P) 2.02 1.16-3.51 Paper survey (N) OR 0.50, 95% CI 0.34-0.75 | X |
| 4 | 39 | Bhuyan | 2016 | Higher education (P) Health insurance (P)  Confident in self-care (P) Comorbidities (P) Older (N) Higher income (N) Living in rural areas (N)  Increase in age (N) |  |  |  | Individual (Having  mHealth apps) | Odds ratios for access and adoption | Higher education (college or more vs high) (P) 4.65*** [2.29, 9.47] Health insurance (P) 1.67* [1.05, 2.64] Confident in self-care (P) 1.46* [1.08, 1.97] Comorbidities (2 or more) (P)1.49* [1.04, 2.15] Older (65+ vs younger than 35) (N) 0.23*** [0.15, 0.34] Higher income (N) 0.58* [0.34, 0.99] Living in rural areas (N) 0.67* [0.45, 1.00]  Female 1.09 [0.82, 1.45]  African American 1.14 [0.82, 1.58]  Hispanic ethnicity 0.76 [0.53, 1.09]  Regular provider 1.34 [0.97, 1.85]  Unemployed 1.14 [0.84, 1.54]  Poor health status 1.29 [0.78, 2.14]  Severely obese 0.41 [0.09, 1.89]  Current smoker 1.21 [0.79, 1.85] | X |
|  |  |  |  |  | Older (P) Severely obese (P) Higher income (N) | Black (P)  Confidence in self-care (P) |  | Individual / Interpersonal  (Use of mHealth  apps for achieving health behavior goals) |  | Older (P) 0.13*** [0.05, 0.31] Severely obese (vs underweight) (P) 7.03* [1.47, 33.63] Higher income (N)0.40* [0.17, 0.97] Higher education (college or more vs high) 0.91 [0.20, 4.12] Confident in self-care 0.80 [0.44, 1.44]  Current smoker 0.87 [0.45, 1.69]  Female 1.41 [0.82, 2.41]  African American 0.88 [0.45, 1.74]  Hispanic ethnicity 0.97 [0.48, 1.95]  Regular provider 0.77 [0.41, 1.43]  Unemployed 1.23 [0.64, 2.38]  Health insurance 1.52 [0.70, 3.31]  Living in rural areas 1.16 [0.62, 2.15]  Poor health status 0.74 [0.24, 2.29]  Current smoker 0.87 [0.45, 1.69]  Comorbidities (2 or more) 1.55 [0.81, 2.97] |  |
| 5 | 84 | Bishwajit | 2017 |  | Non-slum area (P) No education (N) Richer (P)  Postnatal care for newborns (P) |  |  | Individual | Odds ratios for adoption | Non-slum: 1.282 (1.076–1.526) No education: 0.639 (0.486–0.839) Richest: 4.108 (3.145–5.367)  Postnatal care for respondents: 0.650 (0.406–1.039)  Postnatal care for newborns: 2.707 (1.712–4.279) | X |
| 6 | 54 | Bommakanti | 2020 | Older age (P) Male (P) Lower income (P) Lower educational attainment (P) |  |  |  | Individual | Odds ratios for access | Older age: 1.09 (1.05, 1.13) Male: 2.86 (1.04, 7.86) <$10,000: 3.06 (1.19, 7.89) High school or below: 4.48 (1.57, 12.80) | X |
| 7 | 40 | Bonnell | 2022 | Language barrier (N) Diverse health literacy (N) Low digital literacy/numeracy (N) Unmet preferences for accessing information (N) | Limited experience (N) Concerned about data security, privacy (N) Mistrust of government, law enforcement, and technology companies (N) Fear of negative consequences from engaging with exposure notification tools (N) |  |  | Community | N/A |  |  |
| 8 | 69 | Buss | 2022 |  | At risk of cardiovascular disease or type 2 diabetes mellitus (N) Male (N) Older (N) Physically diabled (N) Lower income (N) |  |  | Individual | Odds ratios for adoption | At risk of cardiovascular disease or type 2 diabetes mellitus (N) 1.06, 95% CI 0.97-1.16; Male (N) 0.74 (0.68, 0.79) Older (N) 0.94 (0.93, 0.94) Physically diabled (N) 0.78(0.72, 0.84) Lower income (N) 0.40(0.35, 0.46) | X |
| 9 | 70 | Camacho-Rivera | 2020 |  | Younger age (N) Female (P) Higher education (P) White (N) At least 1 COVID-19 symptom (P) Employed (N) Lower household income (N) |  |  | Individual | Prevalence ratios for adoption | Younger age (N) 0.71 (0.59-0.86) Female (P) 1.12 (1.01-1.24) Baccalaureate (vs high school) (P) 0.75 (0.65-0.87) White (N) (vs Black) 1.58 (1.36-1.83) At least 1 COVID-19 symptom (P) 1.13 (1.02-1.25) Employed (N) 0.85 (0.76-0.95) Lower household income (N) 0.82 (0.73-0.93) |  |
| 10 | 71 | Cao | 2022 |  | Digital access (P) Digital use (P) Employed (P) |  |  | Individual | Regression weights of parameters (SEM) | Digital access (P) 0.28 (95% CI 0.22-0.34) Digital use (P) 0.51 (0.38 to 0.64) Employed (P) 0.08 (0.02 to 0.15) |  |
| 11 | 72 | Che Johan | 2023 |  | Readiness and ability to use mHealth (P) |  |  | Individual | Correlation coefficients | Ability and readiness to use mHealth apps (r=0.4; P<0.05) |  |
| 12 | 73 | Chen | 2023 |  | Black (P) Hispanic (N) Shared decision-making with HCPs (P) |  |  | Individual | Odds ratios for adoption | Any mHealth usage Black: 1.62 (1.13-2.32) Hispanic: 0.69 (0.50-0.97)  Non-Hispanic Asian 1.26 (0.73-2.20)  Non-Hispanic other 1.67 (0.86-3.22)  by SDM Black (always SDM): 1.88 (1.16- 3.05) Hispanic (not always SDM): 0.52 (0.31- 0.88) Non-Hispanic other (always SDM): 3.12 (1.23- 7.93) | X |
| 13 | 85 | Choudhury | 2023 |  | Perceived usefulness (P) Self-care ability (P) Severity of conditions (ID-U) (P) Education (ID-U) (P) Perceived ease of use (ID-U,SC-A) (P) Self-efficacy (ID-IDM) (P) Age (ID-IDM) (N) |  |  | Individual | Beta (SEM) | Perceived usefulness: 0.319 Self-care ability : 0.196 Severity of conditions (ID-U): 0.413 Education (ID-U): 0.135 Perceived ease of use (ID-U,SC-A): 0.202 Self-efficacy (ID-IDM): 0.316 Age (ID-IDM): -0.172 |  |
| 14 | 86 | Cilliers | 2017 |  | Attitude toward technology (N) Mobile experience (P) Perceived usefulness (P) Social influence (P) |  |  | Individual/ Interpersonal | Beta (EFA, CFA) | Attitude toward technology: -0.16 Mobile experience: 0.211 Perceived usefulness: 0.387 Social influence : 0.19 |  |
| 15 | 55 | Doyle | 2021 | Increase in age (P) Living in apostolic sect (N) Higher education (P) No orphan (P) |  |  |  | Individual/ Community | Odds ratios | Increase in age: 1.40 (1.30-1.52) Living in apostolic sect: 0.46 (0.21-0.99) Higher education (secondary): 2.27 (1.08-4.77)  Tertiary education: 2.64 (0.58-11.94) No orphan: 1.89 (1.07-3.34)  Female: 0.73 (0.49-1.07)  Never married: 1.67 (0.87-3.18)  Divorced/separated: 0.64 (0.15-2.77)  Living with no religion: 0.42 (0.17-1.03)  Employed: 2.24 (0.78-6.45)  Unemployed: 1.09 (0.70-1.69)  Living longer at a community: 1.05 (0.64-1.71) | X |
| 16 | 41 | Ernsting | 2017 | Younger (P) Higher education (P) Use of the Internet for research about health issues (P) Part-time or unemployed (N) More physical activity (P) Low fat diet (N) Higher QoL (P) Health literacy (P) | Younger (P) Use of the Internet for research about health issues (P) Speaking other than mother tongue (P) Many chronic conditions (P) More physical activity (P) Low fat diet (N) Health literacy (P) |  |  | Individual | Odds ratios for access and adoption | <Access> Younger (P) 0.92 (0.91-0.93) Higher education (P) 1.69 (1.2-2.37) Use of the Internet for research about health issues (P) 3.24 (2.6-4.03) Part-time or unemployed (N) 0.60 (0.46-0.79) or 0.48 (0.33-0.7) More physical activity (P) 1.26 (1.03-1.52) Low fat diet (N) 0.67 (0.55-0.81) Higher QoL (P) 1.24 (1.02-1.5) Health literacy (P) 1.05 (1.04-1.07)  Female (vs male) 0.92 (0.76-1.1)  Comorbidities (Three or more) 0.78 (0.49-1.25)  Smoking 1.11 (0.9-1.35)  Adherence (doctor’s advice) 0.96 (0.72-1.27)  <Adoption> Younger (P) 0.97 (0.96-0.98) Use of the Internet for research about health issues (P) 1.68 (1.37-2.06) Speaking other as mother tongue (vs German) (P) 1.5 (1.11-2.02) Many chronic conditions (P) 2.0 (1.07-3.75) More physical activity (P) 1.38 (1.11-1.72) Low fat diet (N) 1.33 (1.06-1.66) Health literacy (P) 1.02 (1-1.03)  Female (vs male) 1.15 (0.94-1.42)  Not working 0.99 (0.61-1.62)  Higher education 1.04 (0.72-1.51)  Smoking 0.98 (0.78-1.22)  Adherence (doctor’s advice) 0.81 (0.57-1.14) | X |
| 17 | 81 | Ernsting | 2019 |  | Younger (P) Female (P) Higher education (P) More physical activity (P) eHealth literacy (P) Wearable use (P) Comorbidities (P) |  |  | Individual | Odds ratios for adoption | <CVD> Younger (P) 0.93 (0.91-0.95) Female (P) 0.68 (0.50-0.94) University degree (P) 8.38 (2.34-30.07) More physical activity (P) 1.78 (1.30-2.43) eHealth literacy (P) 2.52 (1.94-3.28) Wearable use (P) 21.44 (11.6-39.63) DM(P) 1.52 (1.12-2.06)  Stress by CVD (P) 1.29 (1.09-1.51)  Smoking 0.84 (0.61-1.16)  Balanced diet 1.18 (0.83-1.69)  Health literacy 1.10 (0.79-1.53)  <DM> for meta-analysis Younger (P) 0.94 (0.92-0.97) Female (P) 0.64 (0.42-0.98) Higher education (P) 3.53 (1.06-11.75) More physical activity (P) 2.12 (1.40-3.20) eHealth literacy (P) 2.36 (1.69-3.29) Wearable use (P) 12.64 (5.48-29.12) Stress by DM (P) 1.51 (1.23-1.85)  Smoking 0.99 (0.66-1.49)  Balanced diet 1.31 (0.79-2.18)  Health literacy 1.22 (0.79-1.89) | X |
| 18 | 74 | Fradkin | 2022 |  | Female (P) Age 35-54 years (P) Non-Hispanic White or multiracial (P) LGBQA (P) Smoking other types of tobacco products (N) |  |  | Individual | Prevalence estimates for adoption |  |  |
| 19 | 96 | Gershoni | 2023 |  | No difference by race Glycemia (P) |  |  | Individual | Beta (linear) | Frequency of measures REM: -4.38 (1.67) WP: -3.77 (1.02) |  |
| 20 | 82 | Ginossar | 2021 |  | Curiosity (P) Limited connectivity (N) Low self-efficacy (N) Low digital literacy (N) |  |  | Individual/ Community | N/A |  |  |
| 21 | 75 | Hamilton | 2018 |  | Hispanics (N) Spanish-only speaking (N) Low income (N) Higher education (P) Private insurance (P) |  |  | Individual/ Societal | Odds ratios for adoption | Hispanics (N) 0.29 (0.15-0.56) Spanish-only speaking (N) 0.16 (0.08-0.32) Low income (N) 0.25 (0.08-0.72) Higher education (P) 8.03 (3.39-18.99) Uninsured (vs Private insurance) (P) 0.21 (0.08-0.55)  Black 1.10 (0.49, 2.44)  Other race 1.48 (0.28, 7.91)  Older age 0.5 (0.11, 2.3) | X |
| 22 | 91 | Hardy | 2022 |  |  | Frequent use of smartphones (P) More confident in their use prior to therapy (P) |  | Individual | t-value | Frequent use of smartphones: -2.48 (p=0.02) More confident in their use prior to therapy: -2.17 (p=0.03) |  |
| 23 | 42 | Haro-Ramos | 2023 | Latinx (N) | Latinx (N) | Latinx (N) (usability) | Latinx (N) (not easy to continue) NLW (N) (willingness to use in the future and to recommend the program to others) | Individual/ Community | t-test chi-squared test |  |  |
| 24 | 43 | Hengst | 2023 |  | Older (P) Higher education (P) Higher intention and adoption (P) | Older age (P) Previous adoption (P) |  | Individual | Cluster analysis |  |  |
| 25 | 99 | Idris | 2022 |  |  |  | Not having a coach (N) Higher education (P) Neighborhood with higher morbidity and mortality rate related to CVD (P) | Individual | Hazard ratios for maintenance | Not having a coach (N) 0.63 (0.41-0.98) Higher education (P) 3.18 (1.10-9.16) Neighborhood with higher morbidity and mortality rate related to CVD (P) 2.00 (1.03-3.87) |  |
| 26 | 44 | Jiwani | 2023 | Female (P) Young adults (P) College education (P) White (P) Black (N) Latinx (N) | Young adults (N) College education (P) Female (N) Asian (N) Native American or Pacific Islander (N) | College education (P) Female (N) Married (P) Asian (N) Native American or Pacific Islander (N) |  |  | Odds ratios  (only point estimates for adoption and maintenance) | <adoption>  Age (18–34) 0.95  College 1.62  Female 0.87 Married or domestic partnership 1.03  Race African American 0.94  Race Asian 0.87  Race Latinx 1.02  Race Native American or Pacific Islander 0.76  Race other 0.98  <maintenance>  Age (18–34) 0.97  College1.77  Female0.85  Married or domestic partnership 1.05  Race African American0.94  Race Asian 0.85  Race Latinx1.05  Race Native American or Pacific Islander 0.68  Race other 1.01 |  |
| 27 | 52 | Khatun | 2015 | Younger (P) Male (N) Higher education (P) Richer (P) Awareness about mHealth (P) Duration of mobile phone use (P) |  |  |  | Community | Odds ratios for access | 18–29 (vs 50+) (P) 3.8 (3.1–4.7) Male (P) 3.9 (3.4–4.5) Higher education (P) 4.8 (2.7–8.5) Richer (P) 10.1 (7.9–13.0) Awareness about mHealth (P) 2.0 *1.5-2.7) Duration of mobile phone use (P) (5y+ vs <1y)2.7 (1.8-3.9) | X |
| 28 | 45 | Kim | 2015 | Presence of family members (P) Using Wi-Fi at home and public places (P) Navigation difficulties (N) | Lack of skills to use apps (N) Information overload (N) Health literacy (lack of skills in evaluating health information, difficulty in language and comprehension) (N) |  |  | Individual/ Community | N/A |  |  |
| 29 | 76 | Kim | 2021 |  | Intentions to use health apps (with attitudes, injunctive norms, perceived behavioral control) (P) |  |  | Individual | Indirect effects | Attitudes (β=.220, P<.001), PBC (β=.461, P<.001), and injunctive norms (β=.186, P<.001) were positively associated with intentions to use health apps, which, in turn, were positively related to actual use of health apps (β=.106, P=.03). Income was positively associated with intentions to use health apps, and this relationship was mediated by attitudes (B=0.012, 95% CI 0.001-0.023) and PBC (B=0.026, 95% CI 0.004-0.048). |  |
| 30 | 87 | Klaver | 2021 |  | Privacy risk, performance risk, and legal concern about mHealth apps (N) Trust in mHealth apps (P) |  |  | Individual | Beta (linear) | Privacy risk (N) –0.103 (–0.195 to –0.011) performance risk –0.337 (–0.450 to –0.225) legal concern about mHealth apps (N) –0.136 (–0.235 to –0.038) Trust in mHealth apps (P) 0.555 (0.422 to 0.687) |  |
| 31 | 46 | Laing | 2018 | Awareness of medical-based apps Age (N) Washington DC (P) Female (P) | <Use of smartphone for wellness> Age (N) Part-time employment (N) <Use of health apps> Age (N) Higher income (P) <Use of medical-based apps> Hispanic/Latino (P) |  |  | Individual | Odds ratios for access adoption | Awareness of medical-based apps (access) Age (N) 0.95 (0.91, 0.99) Washington DC (P) 3.77 (1.09, 13.01) Female (P) 3.53 (1.35, 9.20)  Higher education 1.38 (0.52, 3.66)  Higher income 1.45 (0.47, 4.44)  Part-time employment 2.57 (0.70, 9.49)  Unemployed 0.75 (0.19, 2.93)  Hispanic 1.49 (0.41, 5.41)  Black 2.78 (0.72, 10.74)  Multiple races 1.30 (0.26, 6.42)  <Use of smartphone for wellness> Age (N) 0.94 (0.88, 0.99) Part-time employment (N) 0.17 (0.03, 0.83)  Female 1.63 (0.47, 5.66)  Higher education 0.70 (0.22, 2.27)  Higher income 1.33 (0.31, 5.78)  Unemployed 0.25 (0.05, 1.37)  Hispanic 1.19 (0.22, 6.35)  Black 0.65 (0.12, 3.57)  Multiple races 0.42 (0.05, 3.45)  <Use of health apps> (adoption) Age (N) 0.95 (0.91, 0.99) Higher income (P) 3.13 (1.02, 9.57)  Female 2.55 (0.97, 6.73)  Higher education 0.83 (0.33, 2.10)  Part-time employment 1.24 (0.36, 4.27)  Unemployed 0.69 (0.19, 2.51)  Hispanic 0.76 (0.21, 2.72)  Black 0.52 (0.14, 2.00)  Multiple races 0.22 (0.04, 1.28)  <Use of medical-based apps> Hispanic/Latino (P) 6.38 (1.04, 39.02)  Age 0.99 (0.96, 1.02)  Female 2.46 (0.68, 8.98)  Higher education 0.76 (0.24, 2.39)  Higher income 1.95 (0.46, 8.37)  Part-time employment 0.61(0.13, 2.91)  Unemployed 0.77 (0.15, 4.06)  Black 1.78 (0.33, 9.78)  Multiple races 0.51 (0.08, 3.39) | X |
| 32 | 63 | Leziak | 2021 |  | Education that reduces uncertainty (P)  Support communities (P) Visualizing progress (P) Convenient access to information (P) Support for better management of pregnancy- or DM- related tasks (P) Personalization (P) Interactive features (P)  Integrated graphics (P) |  |  | Individual | N/A |  |  |
| 33 | 47 | Luo | 2021 | Openness to health apps (P) Comfort using smartphones (P) Current or previous phone usage (P) Knowledge about apps (P) | Concerns about app use (N) Comfort using smartphones (P) |  |  | Individual | N/A |  |  |
| 34 | 95 | Maglalang | 2017 |  | Culturally tailored support (P) |  |  | Individual/ Societal | N/A |  |  |
| 35 | 25 | Mahmood | 2019 | Older (N) Female (P) Employed (P) | Employed (P)  Self-rated general health status (good to poor) (vs excellent or very good) (N) |  |  | Individual/ Interpersonal | Odds ratios for access and adoption | <Access> Older (N) 0.24 (0.08–0.71) Female (P) 1.55 (1.01–2.37) Employed (P) 1.97 (1.06–3.66)  Non-Hispanic Black 1.18 (0.58–2.41)  Hispanic 1.75 (0.66–4.66)  Non-Hispanic Asian and others 2.34 (0.86–6.35)  Higher income 1.71 (0.59–4.96)  Living in urban location 0.67 (0.32–1.41)  Former smoker 1.50 (0.69–3.27)  Never smoker 1.05 (0.49–2.25)  <Adoption> Employed (P) 2.35 (1.12–4.96) Self-rated general health status (good to poor) (vs excellent or very good) (N) 0.36 (0.18–0.73)  Older 0.54 (0.17–1.69)  Female 1.57 (0.96–2.57)  Married 1.69 (0.71-4.03)  Divorced/separated 1.01 (0.35–2.91)  Former smoker 1.14 (0.42–3.07)  Never smoker 0.65 (0.23–1.80)  Overweight 1.11 (0.50–2.44) Fair or poor health status 0.50 (0.20–1.24) | X |
| 36 | 88 | Marhefka | 2020 |  | Older (N) Non-hispanic Black (N) Hispanic (N)  Living in rural area (N) |  |  | Individual | Odds ratios for adoption | Older (65+ vs 18 to 29) (N) 0.06 (0.03, 0.12) Non-Hispanic Black (N) (vs NHW) (0.46 (0.31, 0.69) Hispanic (N) 0.60 (0.38, 0.96)  Living in rural area (N) 0.38 [0.18, 0.81]  Female 1.09 [0.82, 1.44]  Transgender 1.43 [0.40, 5.14]  Non-Hispanic other 0.27 [0.05, 1.66] | X |
| 37 | 48 | Marrie | 2019 | Female (P) Older (N) Higher income (P) Single (P) Severe disability (N) Smoker (N) Physical activity (P) Comorbidity (P)  Online responder (P) | Older (N) Post-graduate degree (P) Higher income (P) Physical activity (P) Comorbidity (P)  Online responder (P) |  |  | Individual | Odds ratios for access and adoption | <Access> Female (P) 1.54 (1.28, 1.87) Increase in age (N) 0.95 (0.94, 0.96) Higher income (P) 4.92 (3.09, 7.83) Single (P) 1.26 (1.05, 1.52) Severe disability (N) 0.75 (0.59, 0.95) Smoker (N) 0.69 (0.53, 0.91) Physical activity (P) 1.26 (1.06, 1.50) Comorbidity (3 or more) (P) 1.30 (1.04, 1.62)  Online responder (P) 2.18 (1.83, 2.59)  Non-white 0.98 (0.77, 1.25)  Post-graduate degree 1.16 (0.92, 1.46) No insurance 1.01 (0.50, 2.06)  Private and public insurance 1.18 (0.92, 1.53)  Only public insurance 1.10 (0.87, 1.38)  Employed 1.28 (0.95, 1.72)  <Adoption> Increase in age (N) 0.967 (0.96, 0.97) Post-graduate degree (P) 1.36 (1.14, 1.62) Higher income (P) 1.45 (1.04, 2.01) Physical activity (P) 1.36 (1.18, 1.55) Comorbidity (P) 1.57 (1.33, 1.85)  Online responder (P) 2.60 (2.17, 3.12)  Female 1.09 (0.94, 1.27)  Non-white 1.01 (0.85, 1.20)  No insurance 0.54 (0.29, 1.01)  Private and public insurance 1.01 (0.84, 1.22)  Only public insurance 0.92 (0.77, 1.09)  Employed 1.06 (0.89, 1.27)  Severe disability 0.83 (0.66, 1.05) Smoker 0.83 (0.66, 1.05) | X |
| 38 | 98 | Meijer | 2021 |  | Professional coach (P) Enabled push notifications (P) |  |  | Individual | Odds ratios for adoption | Professional coach (P) 4.06 (1.94–8.50) Enabled push notifications (P) 2.18 (0.92–5.18)  Previous quit attempts 1.45 (0.77–2.74)  Quitting smoking for longevity 0.90 (0.42–1.94) | X |
| 39 | 89 | Melhem | 2023 |  | Living in urbanized regions (P) Low digital literacy (N) |  |  | Individual | Odds ratios for adoption | Living in urbanized regions (P) 13.285 (1.793, 98.414) Low digital literacy (very poor vs very good) (N) 0.013 (0.002, 0.112)  Male 1.469 (0.413, 5.233)  Older age 3.353 (1.039, 10.824)  Higher education 1.113 (0.127, 9.767)  Lower income 2.061 (0.362, 11.718) | X |
| 40 | 61 | Miller | 2017 | Texting <3 days per week (P) No Internet use in the past 30 days (P) Age >57 years (P) |  |  |  | Individual | Odds ratios for access | Texting <3 days per week (P) 3.74 (1.12-12.5) No Internet use in the past 30 days (P) 3.63 (1.19-11.1) Age >57 years (P) 3.69 (1.39-9.80)  No cell phone ownership 1.08 (0.41-2.87)  Limited health literacy 1.33 (0.57-3.10)  Black race 1.19 (0.50-2.87)  Annual income < US $20,000 1.01 (0.37-2.77)  Male gender 0.93 (0.41-2.14) | X |
| 41 | 56 | Moon | 2022 | Age (N) More years of full-time education (P) Living in deprived areas (N) |  |  |  | Individual / Community | Odds ratios for access | Age (N) 0.93 (0.91–0.94) More years of full-time education (P) 1.10 (1.04–1.16) Living in deprived areas (N) 0.40 (0.26–0.61)  White British 0.88 (0.49–1.56) | X |
| 42 | 92 | Nelson | 2016 |  |  | <Text> Age 25 until roughly age 50 years (P) Age after 50 (N) More depressive symptoms (N)  <IVR call> Non-White (N) Lower health literacy (N) |  | Individual | Odds ratios (Non-linear) for adherence | Non-Whites had a 63% decreased relative odds (adjusted odds ratio [AOR] 0.37, 95% confidence interval [CI], 0.19-0.73) of participating in calls.  Lower health literacy 0.67, 95% CI, 0.46-0.99  Older age (nonlinear P=.01)  More depressive symptoms 0.62, 95% CI, 0.38-1.02 |  |
| 43 | 77 | Nelson | 2020 |  |  | Black (vs White) (N) Worse baseline medication adherence (N) Worse HbA1c (N) |  | Individual | 6-month response rate, estimate | Black (vs White) (N) –0.070 (–0.129 to –0.012) Worse baseline medication adherence (N) 0.014 (0.006 to 0.023) Worse HbA1c (N) –0.019 (–0.036 to –0.004) |  |
| 44 | 49 | Nelson | 2022 | Lower income (N) Limited health literacy (N) | Older (N) Lower education (N) Lower income (N) Limited health literacy (N) |  |  | Individual | Odds ratios for access and adoption | <Access> Lower income (N) 0.390 (0.175, 0.870) Limited health literacy (N) 0.497 (0.274, 0.900)  Older age 0.600 (0.318, 1.131) Gender (male) 0.925 (0.507, 1.685)  Non-Hispanic Black 1.002 (0.527, 1.905)  Lower education 0.562 (0.302, 1.045)  Underinsured 0.988 (0.511, 1.913)  Hemoglobin A1c ≥8.5% 0.859 (0.466, 1.583)  <Adoption> Older (N) 0.222 (0.120, 0.411) Lower education (N) 0.424 (0.209, 0.858) Lower income (N) 0.578 (0.343, 0.976) Limited health literacy (N) 0.343 (0.181, 0.650)  Gender (male) 0.795 (0.450, 1.406)  Non-Hispanic Black 0.899 (0.451, 1.791)  Underinsured 0.804 (0.394, 1.642)  Hemoglobin A1c ≥8.5% 0.978 (0.542, 1.766) | X |
| 45 | 78 | Neves | 2021 |  | Single (P) More than 10 years of education (P) Higher digital literacy scores (P) Depression symptoms (N) |  |  | Individual | Odds ratios for adoption | Single (P) 2.22 (1.05-4.75) More than 10 years of education (P) 1.95 (1.12-3.45) Higher digital literacy scores (Q4 vs Q1) (P) 11.52 (4.78-30.87) Depression symptoms (N) 0.32 (0.12-0.83)  Female 1.37 (0.86-2.20)  Older age 1.68 (0.19-11.47)  Separated 1.64 (0.55-4.96)  Deprived 1.34 (0.56-3.24)  Healthier 0.86 (0.42-1.76)  Former smokers 1.83 (0.97-3.53)  Current smokers 0.84 (0.34-2.05)  Anxiety symptoms 1.12 (0.64-1.95)  Uncontrolled asthma 0.89 (0.54-1.44)  Asthma exacerbation 0.99 (0.56-1.75)  Unplanned health appointment 1.01 (0.52-1.91)  Inpatient admission 1.73 (0.37-8.03) | X |
| 46 | 57 | Okano | 2022 | Living close to a health clinic (P) Male (P) Urban residents (P) Older age (N) Living in perverty (N) |  |  |  | Individual | Odds ratios for access | Living close to an health clinic (P) aOR: 1.31, Bayesian 95% highest posterior density [HPD] region: 1.24–1.39 Male (P) 2.37, 95% HPD region: 1.96–2.84 Urban residents (P) 2.66, 95% HPD region: 2.22–3.18 Older age (61+ vs 26-40) (N) 2.68 (2.44, 2.94) Living in poverty (0 vs 3) (N) 0.35 (0.31, 0.40) | X |
| 47 | 60 | Patel | 2022 | Older (N) Male (N) Lower income (N) Higher cognitive abilities (CASI) (P) |  |  |  | Individual | Odds ratios for access | Older per 10y (N) 0.59 [95% CI, 0.48–0.72] Male (N) 0.70 [95% CI, 0.49–0.89] Lower income (N) 0.62 [95% CI, 0.43–0.86] Higher cognitive abilities (CASI) (P) .22 [95% CI, 1.05–1.42] | X |
| 48 | 58 | Perkes | 2023 | 5-year increase in age (N) - smartphone With tertiary education (P) - tablet or laptop |  |  |  | Individual | Odds ratios for access | 5-year increase in age (N) - smartphone (OR 0.723, 95% CI 0.509-0.834; P<.001) With tertiary education (P) - tablet or laptop (OR 1.916, 95% CI 1.095-3.354; P=.02) | X |
| 49 | 53 | Petros | 2022 | Female (P) | Male (P) |  |  |  | Beta (linear) | uMARS 0.16 (0.07; 0.02 to 0.30) (p=0.03) use of the NEVERMIND: −0.13 (0.06; −0.25 to −0.01) (p=0.04) |  |
| 50 | 97 | Pollock | 2023 |  |  | Black (P) Strategies that fit their lives (P) Nonjudgmental communication style (P) Difficult to navigate (N) Insufficient duration of coaching (N) |  | Individual | mean SD |  |  |
| 51 | 90 | Potdar | 2020 |  | Older (N) College-level degree (P) A desire to increase cancer-related knowledge (P) |  |  | Individual | Odds ratios for adoption | Older (N) 0.05, CI 95% 0.01–0.23 College-level degree (vs Less educated) (P) 2.78, CI 95% 1.25–5.88 A desire to increase cancer-related knowledge (P) OR 261.53, CI 95% 10.13–6748.71 | X |
| 52 | 64 | Ramaswamy | 2023 |  | High cost (N) Complexity of use (N) Tutoring (P) |  |  | Individual | N/A |  |  |
| 53 | 93 | Schoenberg | 2021 |  | Personal and supportive connections (P)  Local coaches (P) Educational opportunities (P) |  |  | Individual/ Interpersonal/ Community | N/A |  |  |
| 54 | 50 | Schrauben | 2021 | Older (N) Higher education (P) Higher annual income (P) Adequate health literacy (P) | Older (N) Higher education (P) Higher annual income (P) Depression symptom score (P) <Interest> Non-Hispanic Black (P) Hispanic (P) |  |  | Individual | Prevalence ratios for access and adoption | <Access> Older (N) 0.84 (0.80-0.88) Higher education (P) 1.27 (1.04-1.56) Higher annual income (P) 1.13 (1.00-1.28) Adequate health literacy (P) 1.25 (1.00-1.57) Non-Hispanic Black 1.08 (0.98-1.19)  Hispanic 1.16 (0.97-1.38)  Increase in cognition score 1.05 (0.98-1.13)  Comorbidities 0.96 (0.84-1.10)  Depression symptom score (per 1-SD greater) 1.01 (0.98-1.05)  Disease self-efficacy (per 1-SD greater)  1.03 (0.96-1.11)  Social support (per 1-SD greater) 1.10 (0.99-1.22)  <Adoption> Older (N) 0.71 (0.63-0.80) Higher education (P) 2.62 (1.32-5.17) Higher annual income (P) 1.46 (1.07-2.00) Depression symptom score (P) 1.09 (1.00-1.19)  Non-Hispanic Black 0.89 (0.71-1.10)  Hispanic 1.02 (0.64-1.63)  Adequate health literacy 1.92 (0.98-3.77)  Increase in cognition score 1.04 (0.87-1.25)  Comorbidities 0.98 (0.69-1.39)  Depression symptom score (per 1-SD greater) 1.09 (1.00-1.19)  Disease self-efficacy (per 1-SD greater)  1.11 (0.93-1.32)  Social support (per 1-SD greater) 1.11 (0.90-1.37)  <Interest> Non-Hispanic Black (P) 1.17 (1.08-1.27) Hispanic (P) 1.20 (1.03-1.41) |  |
| 55 | 79 | Shah | 2021 |  | Being married (P) |  |  | Individual | Odds ratios for adoption | Being married (P) 2.12; 95% CI 1.02–4.39  Increase in age 0.98 (0.95–1.01)  Female 0.60 (0.29–1.25)  White race 1.22 (0.60–2.48)  Private insurance 2.62 (0.81–8.46)  Medicare 1.15 (0.29–4.50) | X |
| 56 | 65 | Steinberg | 2022 |  | Greater electronic health literacy (P) Lower patient activation (P) Greater self-efficacy (P) |  |  | Individual | Mean IQR |  |  |
| 57 | 94 | Umaefulam | 2022 |  | Use of Indigenous languages (P) Message content (P)  Appropriate frequency of messages (P) Supportive and encouraging messaging tones (P) |  |  | Individual/ Interpersonal | N/A |  |  |
| 58 | 66 | Yang | 2021 |  | Medicaid beneficiaries (N) <Non-Medicaid> Female (P) Internet access diversity (P) Education (P) Higher household income (P) Cardiovascular disease (P)  Depression (P)  Black (N)  Rural (N)  Non-Hispanic Asian (P) |  |  | Individual/ Societal | Odds ratios for adoption | <Non-Medicaid> Female (P) 1.56 (1.35-1.80) Internet access diversity (P) 1.47 (1.29-1.68) College or higher (vs less than high school) (P) 2.04 (1.38-3.02) Higher household income (P) 2.81 (1.88-4.22) Cardiovascular disease (P) 1.44 (1.20-1.72)  Depression (P) 1.41 (1.18-1.68)  Hispanic 0.92 (0.69-1.22)  Non-Hispanic Black (N) 0.69 (0.51-0.95)  Non-Hispanic Other 1.10 (0.65-1.86)  Non-Hispanic Asian 1.59 (P) (1.04-2.44)  Older age 1.18 (0.76-1.82)  Rural (N) 0.71 (0.55-0.90) | X |
| 59 | 51 | Ye | 2021 |  | <Wearables> Increase in age (P)  Female (P) Black (P) Other race (P) Higher income (P) Higher education (P) Being married or near marriage (P) Higher BMI (P) |  |  | Individual | Odds ratios for adoption | <Wearables>  Increase in age (P) 0.95 (0.95,0.96) Female (P) 1.48 (1.35,1.62) Black (P) 1.34 (1.18, 1.52) Other race (P) 1.30 (1.09, 1.55) Higher income (P) 2.48 (2.11,2.92) Higher education (postgraduate vs high school or lower) (P) 2.05 (1.78, 2.35) Being married or near marriage (P) 1.39 (1.23,1.58) Higher BMI (P) 1.43 (1.28,1.60)  Hispanic 1.05 (0.92, 1.20) | X |
| 60 | 59 | Yepes | 2016 | Female (P) Older (N) Higher SES (P) University-level education (P) Higher annual income (P) Professional occupation (P) |  |  |  | Individual | Odds ratios (only point estimates for access) | <Mobile phone> Female (P) 6.81 Older (N) 0.27 University-level education (P) 7.70 Higher annual income (P) 13.8 Professional occupation (P) 2.31  <Smartphone> Female (P) 1.44 Older (N) 0.15 University-level education (P) 7.42 Higher annual income (P) 1.93 Professional occupation (P) 3.13 |  |
| 61 | 67 | Yu | 2021 |  | Performance expectancy and effort expectancy (P) Family to provide technical support (P) Social support (P) Poor eyesight (N) |  |  | Individual/ Interpersonal/ Community | N/A |  |  |
| 62 | 83 | Żarnowski | 2022 |  | <Mobile apps> Younger (P) Living in large cities (P) Following a diet (P) Regular weight control (P) Minimal physical activity (P) Occasional alcohol consumption (P) Participation in group sports (P)  <Wearables> Younger (P) Good financial status (P) Regular weight control (P) Daily physical activity (P) Physical activity 3-4 times per week (P) Daily alcohol consumption (P) |  |  | Individual | Odds ratios for adoption | [Physical activity] <Mobile apps> for meta-analysis Younger (P) 3.77 (1.84-7.75) Living in large cities (P) 2.14 (1.22-3.74) Following a diet (P) 1.54 (1.04-2.28) Regular weight control (P) 1.76 (1.16-2.67) Regular physical activity (P) 5.58 (2.22-14.04) Occasional alcohol consumption (P) 2.46 (1.19-5.11) (1-2 times per week) Participation in group sports (P) 1.70 (1.04-2.76)  Married 0.66 (0.35-1.26)  Not having children 1.07 (0.64-1.77)  Employed 1.25 (0.79-1.98)  Not having chronic diseases 1.32 (0.88-1.98)  <Wearables> Younger (P) 2.60 (1.53-4.39) Good financial status (P) 1.65 (1.07-2.54) Regular weight control (P) 1.54 (1.10-2.16) Daily physical activity (P) 2.28 (1.27-4.09) Physical activity 3-4 times per week (P) 1.90 (1.05-3.42)  [Diet] <Mobile apps> Younger (P) 7.73 (2.96-20.17) Following a diet (P) 2.71 (1.77-4.14) Regular weight control (P) 2.19 (1.36-3.53) Occasional alcohol consumption (P) 2.52 (1.14-5.58) (2-3 times per month) Participation in group sports (P) 2.29 (1.36-3.87)  Married 1.50 (0.62-3.65)  Not having children 1.69 (0.87-3.30)  Employed 0.94 (0.58-1.53)  Regular physical activity 1.78 (0.76-4.19)  Smoker 1.60 (0.99-2.60)  <Wearables> Regular weight control (P) 3.15 (1.96-5.06) Daily physical activity (P) 3.91 (1.77-8.66) Physical activity 3-4 times per week (P) 4.17 (1.88-9.29) Daily alcohol consumption (P) 3.40 (1.41-8.24) Participation in group sports (P) 1.28 (0.77-2.13) | X |

* (P) and (N) indicates a positive and negative association with statistical significance, respectively.
