# Supplementary material for: Understanding Inequalities in Mobile Health Utilization Across Phases: Systematic Review and Meta-Analysis
Source: J Med Internet Res. 2025 Aug 14;27:e71349. doi: 10.2196/71349 (PMC12352709; doi:10.2196/71349)
Supplement: Multimedia Appendix 5 [file jmir-v27-e71349-s005.docx]

**Multimedia Appendix 6. Forest plot showing synthesized effect sizes of mHealth utilization by race/ethnicity in the access (a) and adoption (b) phase. mHealth: mobile health; OR: odds ratio; CI: confidence interval; CI: confidence interval.**


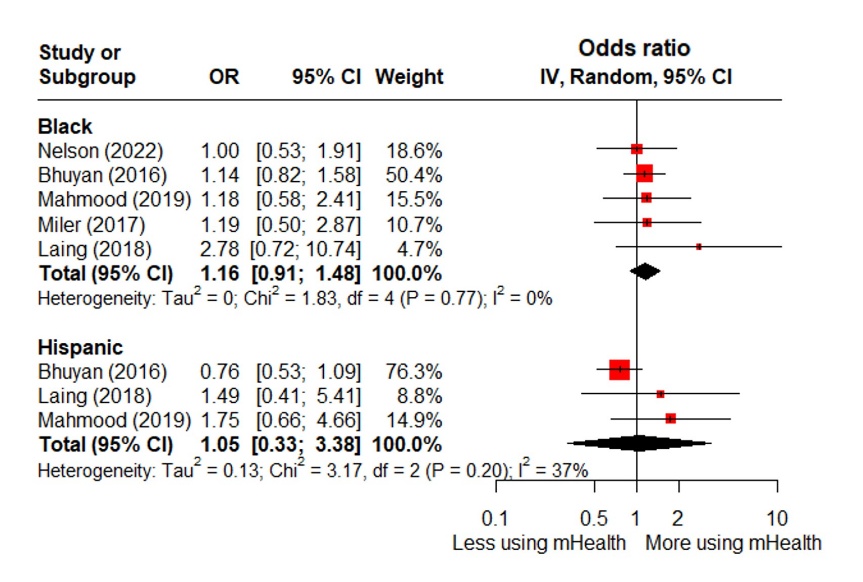


(a)


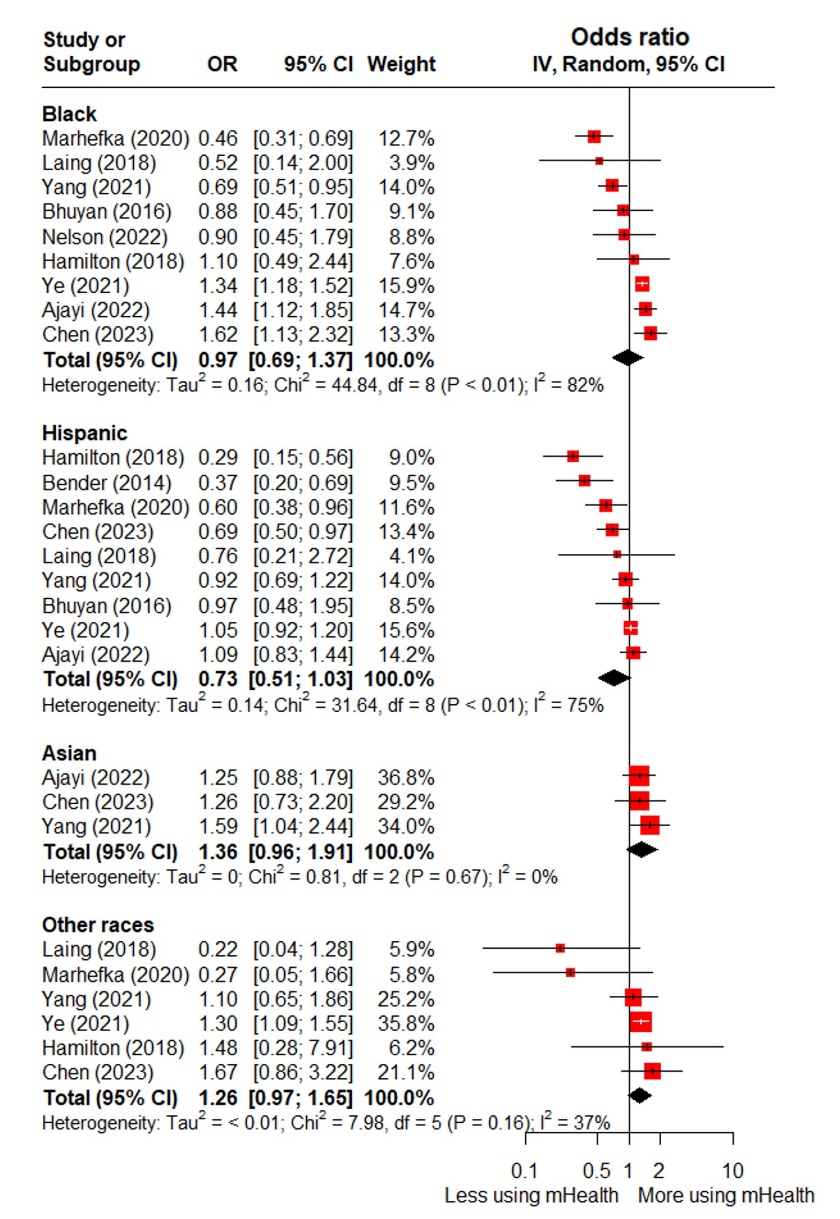


(b)
