# Supplementary figures and images for: Understanding Inequalities in Mobile Health Utilization Across Phases: Systematic Review and Meta-Analysis
Source: J Med Internet Res. 2025 Aug 14;27:e71349. doi: 10.2196/71349 (PMC12352709; doi:10.2196/71349)

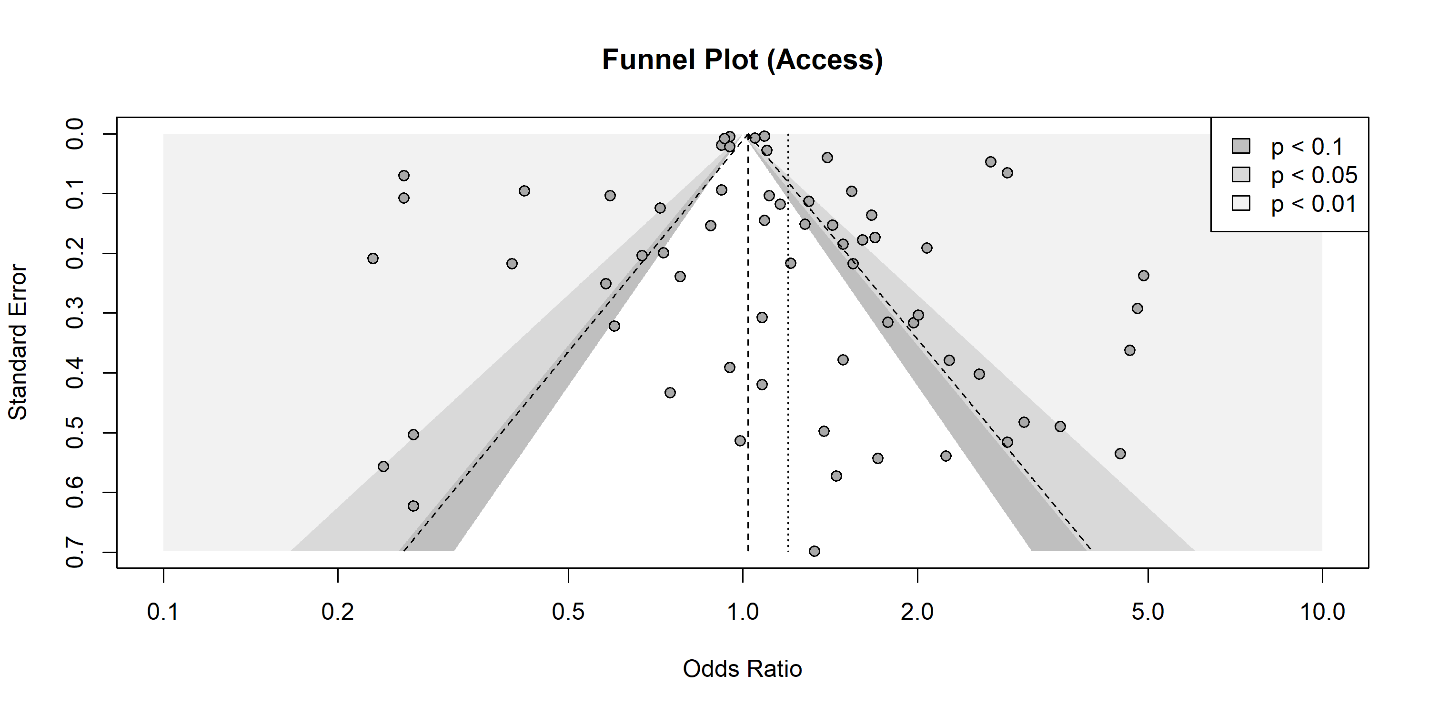


(a)


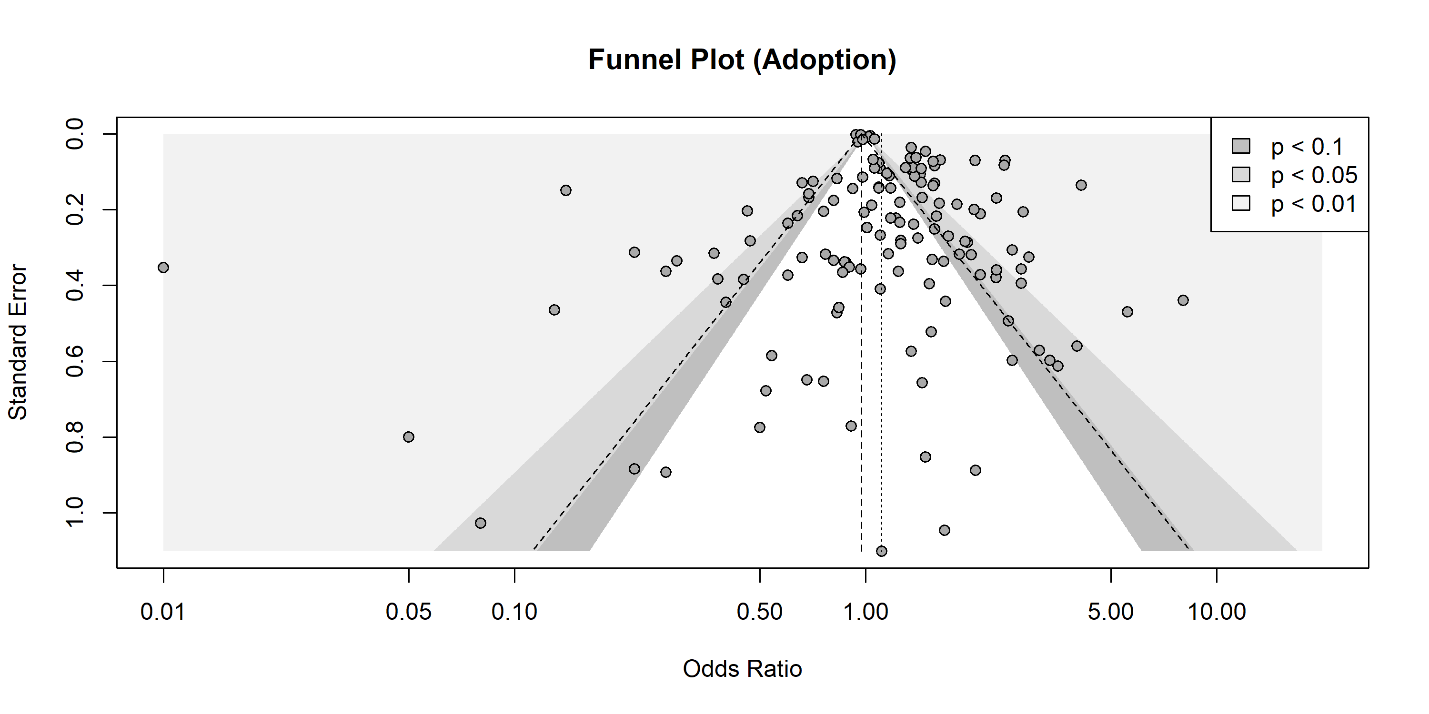


(b)

Supplement: Multimedia Appendix 6 [file jmir-v27-e71349-s006.docx]
